# Mitochondria‐derived vesicles: A promising and potential target for tumour therapy

**DOI:** 10.1002/ctm2.70320

**Published:** 2025-05-12

**Authors:** Xueqiang Peng, Yu Gao, Jiaxing Liu, Xinxin Shi, Wei Li, Yingbo Ma, Xuexin Li, Hangyu Li

**Affiliations:** ^1^ Department of General Surgery The Fourth Affiliated Hospital China Medical University Shenyang China; ^2^ Group of Chronic Disease and Environmental Genomics School of Public Health China Medical University Shenyang China; ^3^ Shenyang Clinical Medical Research Center for Diagnosis Treatment and Health Management of Early Digestive Cancer Shenyang China; ^4^ Department of General Surgery The First Hospital of Anhui University of Science & Technology Huainan China; ^5^ Depatment of Hepatobiliary Surgery Air Force Medical Center Beijing China; ^6^ Division of Genome Biology Department of Medical Biochemistry and Biophysics Karolinska Institutet Stockholm Sweden; ^7^ Department of General Surgery The First Affiliated Hospital of Jinzhou Medical University Jinzhou Liaoning China

**Keywords:** mitochondria‐derived vesicle, target, transport pathway, tumour progression, tumour therapy

## Abstract

Mitochondria‐derived vesicles (MDVs) participate in early cellular defence mechanisms initiated in response to mitochondrial damage. They maintain mitochondrial quality control (MQC) by clearing damaged mitochondrial components, thereby ensuring the normal functioning of cellular processes. This process is crucial for cell survival and health, as mitochondria are the energy factories of cells, and their damage can cause cellular dysfunction and even death. Recent studies have shown that MDVs not only maintain mitochondrial health but also have a significant impact on tumour progression. MDVs selectively encapsulate and transport damaged mitochondrial proteins under oxidative stress and reduce the adverse effects of mitochondrial damage on cells, which may promote the survival and proliferation of tumour cells. Furthermore, it has been indicated that after cells experience mild stress, the number of MDVs significantly increases within 2–6 h, whereas mitophagy, a process of clearing damaged mitochondria, occurs 12–24 h later. This suggests that MDVs play a critical role in the early stress response of cells. Moreover, MDVs also have a significant role in intercellular communication, specifically in the tumour microenvironment. They can carry and transmit various bioactive molecules, such as proteins, nucleic acids, and lipids, which regulate tumour cell's growth, invasion, and metastasis. This intercellular communication may facilitate tumour spread and metastasis, making MDVs a potential therapeutic target. Advances in MDV research have identified novel biomarkers, clarified regulatory mechanisms, and provided evidence for clinical use. These breakthroughs pave the way for novel MDV‐targeted therapies, offering improved treatment alternatives for cancer patients. Further research can identify MDVs' role in tumour development and elucidate future cancer treatment horizons.

## Graphical abstract

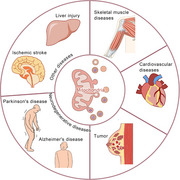

## 1 BACKGROUND

**FIGURE 1:**
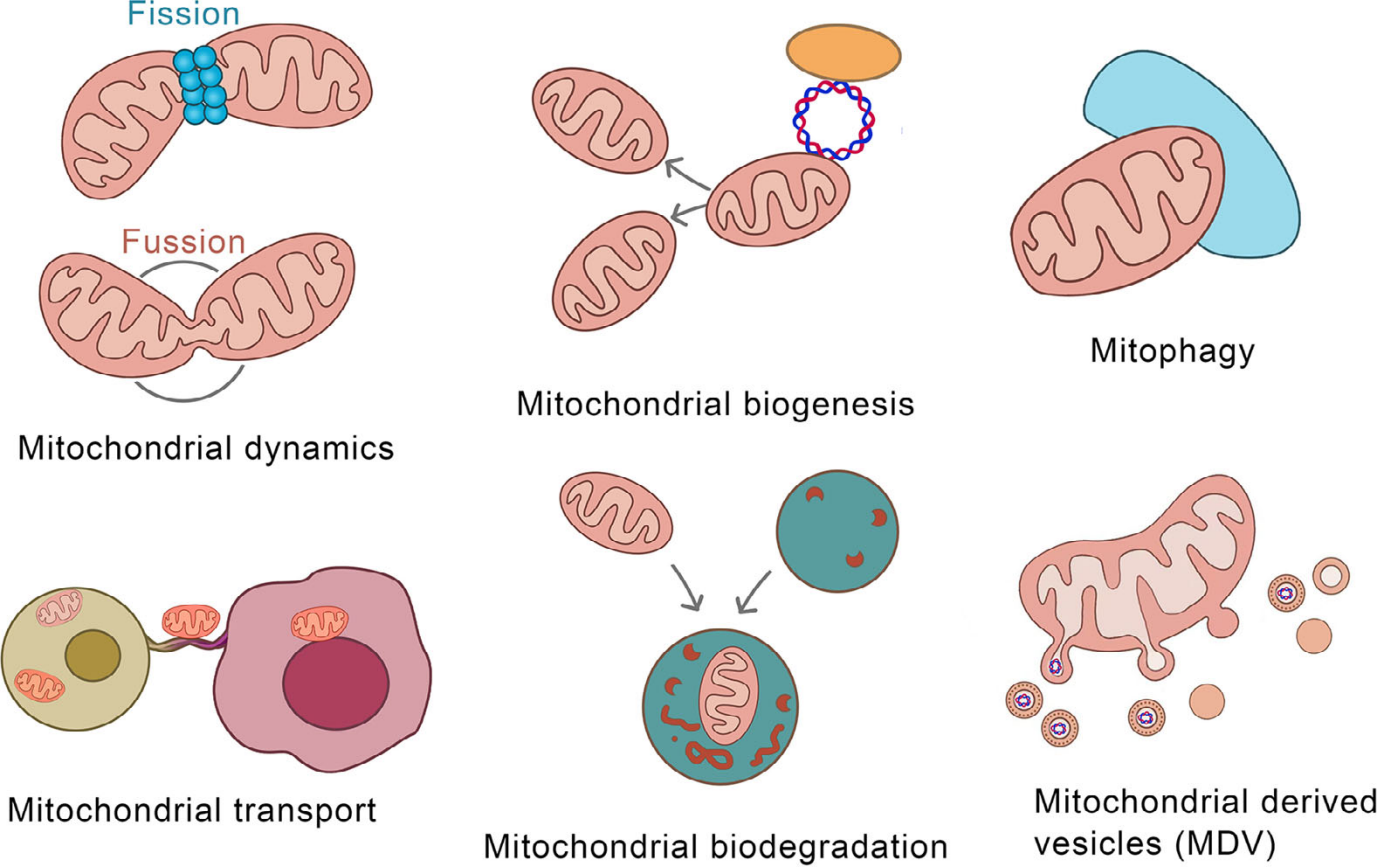
Various mechanisms of mitochondrial quality control (MQC). Mitochondrial quality control comprises a complex network of processes aimed at maintaining mitochondrial health and function. Key components of this control system include mitochondrial dynamics (fission and fusion), mitochondrial biogenesis, mitophagy, mitochondrial trafficking, and mitochondrial proteolysis. The emerging field of mitochondrial‐derived vesicles (MDVs) is pivotal for maintaining mitochondrial homeostasis and cellular health.

**FIGURE 2:**
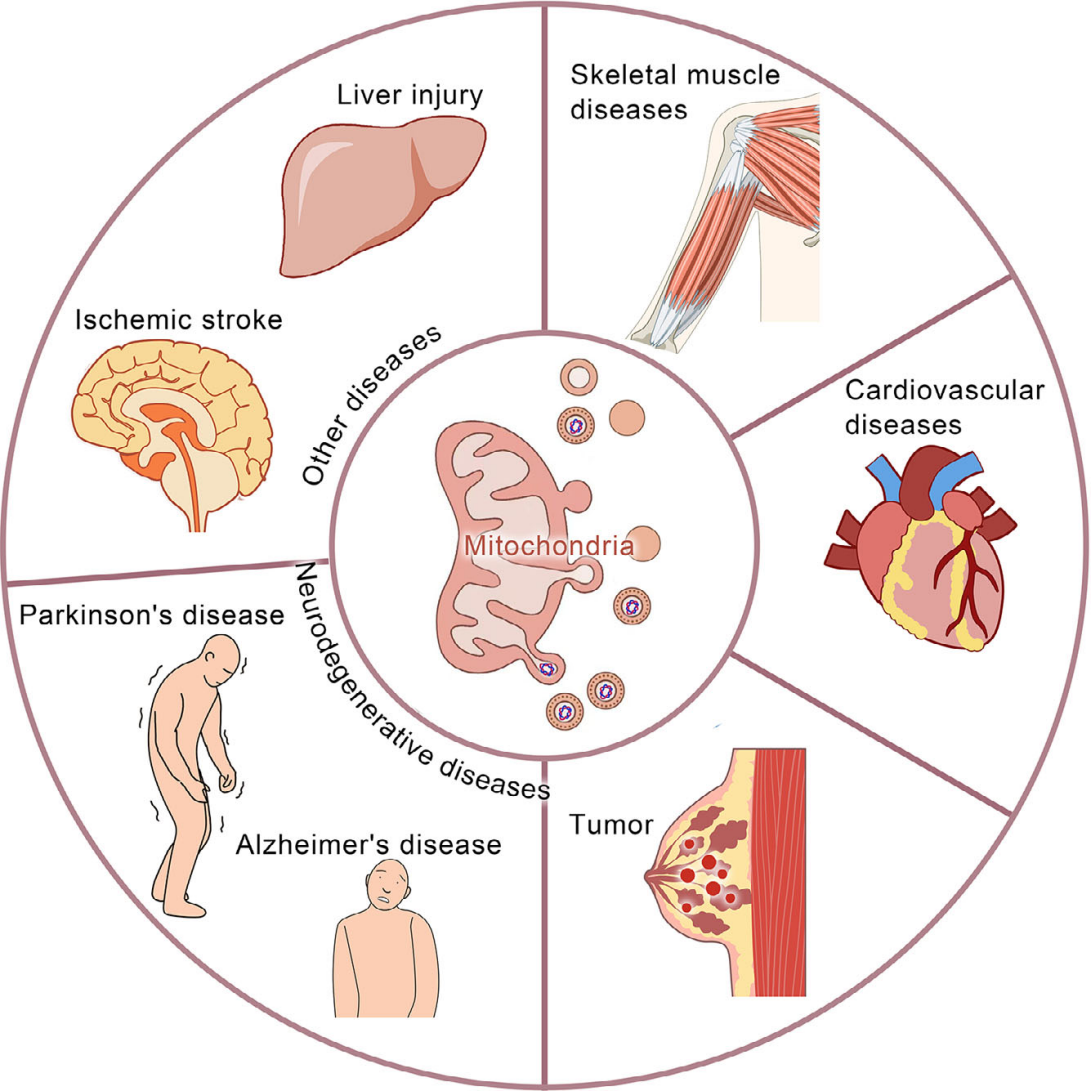
Mitochondrial‐derived vesicles (MDVs) and diseases. MDVs as a mechanism for mitochondrial quality control play a significant role in the development and progression of various diseases, such as tumours, neurodegenerative diseases (such as Parkinson's disease, Alzheimer's disease), cardiovascular diseases, skeletal muscle, etc. Research studies have indicated that MDVs are also involved in the development of diseases such as ischemic stroke and liver injury.

Mitochondria, eukaryotic ubiquities, are central to aerobic respiration,^1^ harbours respiratory enzymes that catalyse ATP synthesis – meeting ∼95% of cellular energy needs.^2^ Beyond bioenergetics, mitochondria regulate proliferation, apoptosis, signalling cascades, and calcium dynamics.^3^ This functional pleiotropy positions mitochondria as master regulators of cellular homeostasis.^4^ Dysfunctional mitochondria disrupt these networks, driving pathophysiological cascades,^5^ thus highlighting mitochondrial fidelity as a cornerstone of organismal viability.^6^ Mitochondrial quality control (MQC) is vital for safeguarding essential cellular processes and ensuring normal mitochondrial function and cellular homeostasis.^7^ This includes mitochondrial dynamics,^8^ biogenesis,^9^ mitophagy,^10^ transport,^11^ intracellular protein degradation,^12^ and mitochondria‐derived vesicles (MDVs)^13^ (Figure 1). Mitochondrial dynamics mainly encompass fission and fusion. Phosphorylated Drp1 translocates to mitochondria, forming a ring‐shaped oligomer for fission‐induced membrane division.^14^ MFN1/2 restructure the outer mitochondrial membrane, enhancing fusion.^15^ OPA1 interacts with inner‐membrane phospholipids to form polymers, promoting inner – membrane and matrix changes for fusion.^16^ PGC‐1α regulates mitochondrial biogenesis.^17^ Mitophagy, a specialised autophagy, recruits damaged mitochondria and ATG proteins to form autophagosomes,^18^ occurring after specific damage.^19^ Mitochondrial transport depends on microtubules, actin filaments, and motor proteins like myosin and dyneins; nanotubes also aid transport.^20^, ^21^ The regulation of mitochondrial transfer and its impact on homeostasis are unclear, despite links between Ca^2+^ levels, volume, and fusion.^22^ The ubiquitin–proteasome system, with mitochondrial proteases and chaperones, degrades dysfunctional proteins for quality control.^23^ Cancer is the most formidable challenge to human health, with persistently high incidence and mortality rates.^24^ There have been certain advancements in traditional cancer treatment modalities such as surgery, radiation, and chemotherapy; however, they are still beset by numerous limitations, highlighting the urgent need for novel therapeutic targets and approaches.^25^ Cancer is predominantly characterised by abnormal cellular growth and can be divided into benign (non‐invasive and non‐metastatic) and malignant (cancerous, invasive, and metastatic) types.^26^, ^27^ Cancer development has multiple stages, which are influenced by both genetic and environmental factors. Treatment options include surgery, radiotherapy, chemotherapy, and others.^28^ Furthermore, it has become the 2nd leading cause of death worldwide.^29^ In recent years, MDVs have been extensively studied, which has increased our knowledge about their critical roles in many diseases^30^ (Figure 2). In 2014, Ayumu Sugiura et al.^31^ published a comprehensive review of MDVs, outlining their discovery and transport mechanisms. However, the review did not elucidate the role of MDVs in tumours. Several studies have indicated the critical involvement of MDVs in tumourigenesis and tumour progression.^32^, ^33^, ^34^ Emerging evidence implicates mitochondrial‐derived vesicles (MDVs) in regulating oncogenic processes, including tumour invasion and metastasis. In breast cancer, MDVs encapsulate mitochondrial DNA (mtDNA) within extracellular vesicles (EVs), activating TLR9‐mediated signalling to drive migratory and invasive phenotypes.^35^ Towers et al. further demonstrated that autophagy‐deficient breast cancer and osteosarcoma cells bypass canonical mitophagy by upregulating mitochondrial fusion – mediated by MFN1/MFN2 dynamins – and amplifying MDV biogenesis. These SNX9‐dependent vesicles shuttle damaged mitochondrial cargo directly to lysosomes or peroxisomes, with vesicle production markedly enhanced under pharmacologic stress. This adaptive rewiring of mitochondrial quality control underscores the metabolic plasticity of cancer cells under autophagy suppression, revealing SNX9 or MDV pathways as tractable targets for therapy‐resistant malignancies. Intriguingly, MDV‐mediated organelle crosstalk may extend beyond oncology, potentially contributing to neurodegenerative disorders marked by mitochondrial dysfunction.^32^ Therefore, MDVs can serve as a promising new target for cancer therapy. This review summarises the current data on the roles of MDVs in tumour development and progression, critically examining their potential as therapeutic targets. Moreover, this study also projects future research trajectories, emphasising the necessity for innovative experimental strategies and technologies to further elucidate the multifaceted functions of MDVs in cancer. By providing evidence for targeting MDVs, this review aims to pave the way for the development of more effective and personalised cancer treatments, thereby advancing the frontiers of oncological research.

## 2 MDVS OVERVIEW: DISCOVERY, FORMATION MECHANISMS, AND DISTINCTIONS WITH EVS

Mitochondria are crucial for cellular function and maintain cellular stability through fusion and fission via DRP1. In 2008, Neuspiel et al.^36^ indicated that MAPL overexpression generates DRP1‐independent mitochondrial fragments, MDVs of 70–150 nm diameter lack cristae and resemble transport vesicles. MDVs selectively include mitochondrial cargo and function autonomously, not requiring ATG5 for formation.^31^ This mechanism parallels primitive bacterial processes, suggesting evolutionary conservation. In 2016, the MDV pathway was observed in Arabidopsis, suggesting its association with higher eukaryotes.^37^ Recent research has revealed that PD‐related genes like Parkin and PINK1 are critical for MDV formation,^38^ **^,^** ^39^ mediate mitophagy‐derived vesicles, and intensify under oxidative stress.^40^ Reactive oxygen species (ROS) activates PINK1 and Parkin, leading to oxidised protein accumulation and vesicle formation.^41^ The synthesis of MDV involves ROS‐induced protein oxidation, phosphatidylcholine oxidation, and PINK1‐Parkin activation. It has been observed that MDVs maintain mitochondrial quality and modulate substance exchange.^13^, ^42^ SNX9 interacts with Rab9, dynamin, AP‐2, and clathrin to regulate MDV formation and release.^43^ Further research is needed to fully understand MDV formation mechanisms. MDVs are vesicles with diameters ranging from 70–150 nm,^36^ originating from mitochondria.^31^ They carry various cargoes including mitochondrial inner and outer membranes, proteins, DNA, RNA, and factors such as PINK1, Parkin, and MAPL.^31^, ^44^ MDVs play crucial roles in maintaining mitochondrial quality control, inter‐organellar communication, signalling, metabolic regulation, and energy metabolism, and can also serve as delivery vehicles.^45^ Their ultimate fate may involve lysosomal degradation, transport to peroxisomes, intracellular reuse, or release and secretion.^45^, ^46^, ^47^ EVs, including exosomes, microvesicles, and apoptotic bodies, have diameters of approximately 30–150 nm, 100–1000 nm, and greater than 1000 nm, respectively.^48^, ^49^, ^50^ EVs carry proteins, nucleic acids, lipids, and metabolic products^51^, ^52^, ^53^, ^54^ and are involved in intercellular communication, immune regulation, metabolic regulation, and disease progression, as well as serving as biomarkers and delivery vectors. Their fate includes uptake by recipient cells, transport and fusion within endosomes, release and function of contents, and intranuclear transport and function.^55^, ^56^, ^57^, ^58^ MDVs interact with late endosomes, forming MDVs‐EVs, which carry mitochondrial proteins and DNA^59^ **^,^** ^60^ and are involved in cell communication, immune regulation, clearance of damaged mitochondria, and regulation of tumour stem cells.^61^, ^62^, ^63^, ^64^ They may enter the circulatory system as biomarkers or be degraded extracellularly^65^ (Table 1). Tim König et al. systematically summarised the primary markers of mitochondrial‐derived vesicles (MDVs), which include TOMM20, MID49/51, MF, DRP1, and phosphatidic acid, among others.^45^ Concerning the detection of MDVs, there are currently two primary methods^61^: (1) morphological observation via transmission electron microscopy to assess MDV morphology and (2) detection based on MDV biomarkers, such as employing immunofluorescent co‐staining or Western blot techniques to detect markers like TOMM20 and DRP1. Effective isolation of MDVs is a prerequisite for their detection. Soubannier et al. successfully extracted MDVs in vivo by digesting mitochondria isolated from the body with proteinase K.^66^

**TABLE 1:** Comparison between MDVs and EVs.

		EVs		
	MDVs	MDVs‐EVs	Other EVs	References
Source	Budding of the mitochondrial inner or outer membrane	MDVs fuse with multivesicular bodies and are released extracellularly through contact with the plasma membrane	Exosomes: multivesicular bodies Microvesicles: cell membrane	^31^, ^65^, ^66^, ^67^, ^68^
Diameter	60–150 nm	70–150 nm	Exosomes: 30–150 nm Microvesicles: 100–1000 nm	^43^, ^47^, ^48^, ^49^
Cargo	Mitochondrial related components (inner membrane, outer membrane, proteins, mtDNA, etc.)	Mitochondrial‐related components (proteins, mtDNA, etc.)	Proteins, nucleic acids, lipids, etc.	^31^, ^43^, ^50^, ^51^, ^52^, ^53^, ^60^, ^62^, ^69^, ^70^
Function	Involved in mitochondrial quality control Involved in interorganellar communication Involved in intracellular signal transduction and metabolic regulation	Intercellular communication (immune regulation, myocardial regulation, neural regulation, regulation of tumour cell malignancy, etc.)	Participate in intercellular cargo and information transmission	^44^, ^60^, ^61^, ^62^, ^63^, ^70^, ^71^, ^72^, ^73^
Fate	Lysosomal degradation Transport to peroxisomes Transport to autophagosome Intracellular reuse Release and secretion	Release into the extracellular space of the cell Can be taken up by receptor cells	Release into the extracellular space of the cell Can be taken up by receptor cells	^44^, ^45^, ^46^, ^54^, ^55^, ^56^, ^57^, ^64^, ^74^

## 3 TRANSPORT PATHWAYS AND CARGO SELECTION OF MDVS

**FIGURE 3:**
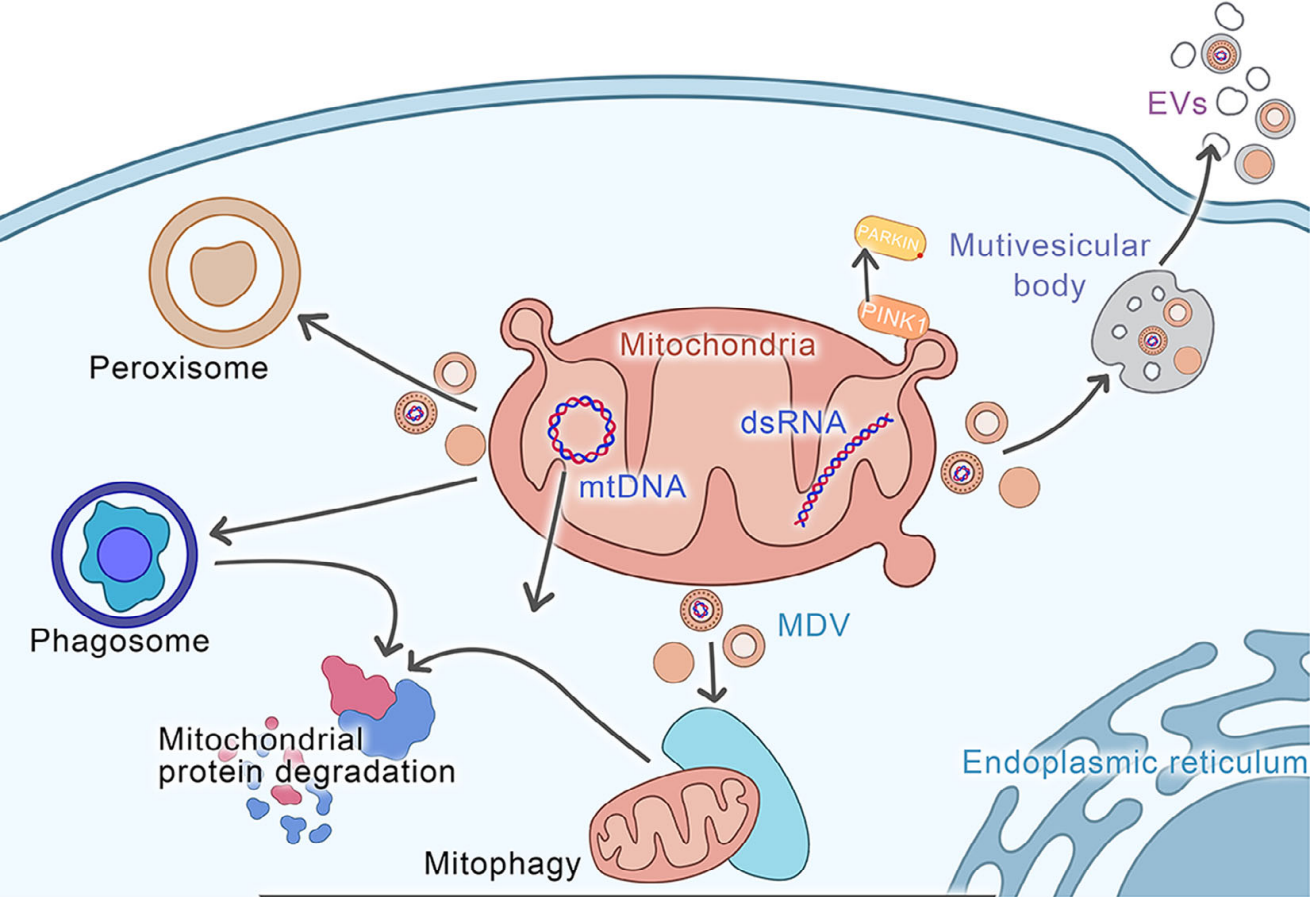
Transport pathways of mitochondria‐derived vesicles (MDVs). The transport pathways of mitochondrial‐derived vesicles (MDVs) are diverse and complex, mainly consisting of three major routes. First, MDVs can be directionally transported to lysosomes. Second, they can target peroxisomes in a specific manner. Third, MDVs can enter multivesicular bodies (MVBs), then be encapsulated into extracellular vesicles (EVs), and finally be secreted into the extracellular environment. Notably, MDVs can not only precisely target exosomes but also, under specific circumstances, be directed towards phagosomes. Among these pathways, the transport route leading to lysosomes is considered the main one for MDVs. In this process, SNARE‐protein‐mediated vesicle fusion, the endosomal sorting mechanism, and the membrane transport regulation involving the VPS35 protein all play crucial roles. This precise and dynamic transport process not only ensures that MDVs can efficiently and selectively reach specific intracellular destinations but also actively participates in the regulation of various cellular functions and is indispensable for maintaining the stability of the intracellular environment. This multipathway and selective transport mechanism enables MDVs to flexibly respond to various signals inside and outside the cell and participate in a series of important physiological processes, including organelle interaction, substance metabolism, signal transduction, and cell communication. Thus, MDVs play a key role in cellular life activities.

Studies have indicated that MDVs utilise two distinct pathways, where one targets the degradation by lysosomes, and the other targets the specific peroxisomal subpopulation.^13^ Furthermore, MDVs also target bacterial phagosomes and extracellular vesicles (EVs)^75^ (Figure 3). Soubannier et al.^66^ revealed that MDVs can selectively enrich oxidised proteins, such as subunits of complex II, III, and IV, but not of complex I and V, and lack any nuclear proteins. This indicates the distinctive densities and uniform diameters of single‐ and double‐membrane vesicles, suggesting their ability to selectively carry cargo. Different types of cargo are selected by each transport pathway^13^ (Figure 3) (Table 2).

**TABLE 2:** Transport pathways and cargo of MDVs.

Selection of cargo	Transport pathway	Mechanism	References
Pyruvate Dehydrogenase Complex(PDH)	Targeting lysosomes	The oxidative stress generated within mitochondria stimulates the entry of different cargo into MDVs, thereby targeting them to the lysosomes.	^76^
VDAC	Targeting lysosomes	The enzyme xanthine oxidase (XO) stimulates cells to produce H_2_O_2_ and induces the formation of single‐membrane MDVs targeted to the lysosomes within mitochondria.	^77^
HOPS complex	Targeting lysosomes	Stx17, in conjunction with SNAP29 and VAMP7, assembles into a ternary complex, mediating MDV‐lysosome fusion.	^78^
TOMM20 protein	Targeting lysosomes	Stx17, together with SNAP29 and VAMP7, forms a ternary complex, facilitating MDV‐lysosome fusion.	^79^
Aberrant endoplasmic reticulum (ER) cargo	Targeting lysosomes	Tollip can interact with the endosomal sorting required for transport‐0 (ESCRT‐0) protein Tom1 within the intracellular system, promoting vesicle maturation.	^80^
TOMM20 protein	Targeting lysosomes	Tollip captures MDVs, facilitates their entry into the endocytic transport system, and recruits cytoplasmic Parkin. After processing and maturation within the endocytic system, cytoplasmic protein Rab7 binds to the vesicle surface, ultimately directing TOM20+ MDVs to the lysosome for degradation.	^41^
TOMM20 protein	Targeting lysosomes	VPS35 recognises MDVs containing the TOM20 protein and associates with the surface oligomers of DRP1, forming TOM20+/ DRP1+ MDVs. Subsequently, the DRP1 oligomers are engulfed by the MDVs, completing the translocation process from the mitochondrial surface to the lysosome for degradation.	^45^
MAPL	Targeting peroxisomes	DRP1 is abundant in MAPL.	^76^
MAPL	Targeting peroxisomes	The ROS generated during xanthine oxidase/xanthine reaction induce the formation of TOM20‐positive single‐membrane MDVs, which selectively transport MAPL to the peroxisomes in HeLa and osteosarcoma cells.	^81^
MAPL	Targeting peroxisomes	The recruitment of vesicle protein sorting‐associated proteins VPS35 and VPS26 forms a complex, which in turn mediates the transport of MAPL to peroxisomes via MDVs.	^62^
DLP1 complex	Targeting peroxisomes	The Vps35 subunit binds to the cargo tail, thereby mediating the transport of MAPL to peroxisomes via MDVs.	^82^
SOD2	Targeting phagosome	Infection of macrophages with methicillin‐resistant *Staphylococcus aureus* stimulates the production of MDVs containing SOD2. The loaded MDVs are transported to phagolysosomes containing the bacteria.	^83^
DAMPS	Targeting EV	Optic atrophy 1 (OPA1) and SNX9 mediate the targeting of MDVs containing DAMPs to extracellular vesicles (EVs).	^63^
Alpha‐synuclein	Targeting EV	When Parkin and PINK1 are mutated, MDVs can load α‐synuclein and enter EVs, facilitating intercellular transfer through EVs.	^84^
mtDNA	Targeting EV	Cancer‐associated fibroblasts can transfer mtDNA through EVs, restoring oxidative phosphorylation in hormonally treated breast cancer cells.	^83^
TOM20 protein	Targeting MVB	MDVs can directly target to multivesicular bodies (MVBs).	^13^
mtDNA	Targeting lysosomes	SNX9 mediates the targeting of mtDNA‐enriched MDVs to the lysosomes.	^34^
mtDNA	Targeting EV	The trafficking of mtDNA in extracellular vesicles facilitates the transmission of oncogenic signals, promoting the therapy‐induced reawakening of cancer stem cell‐like cells.	^64^

### 3.1 Transport pathway targeting lysosomes

The lysosomal pathway is primarily mediated by MDVs containing oxidised proteins and is closely associated with the PINK1/Parkin pathway.^85^ Evidence confirms MDVs selectively transport oxidised products to lysosomes for degradation, aiding mitochondrial stability; however, MDV entrance into lysosomes is complex.^86^ This underscores the intricate MDV‐lysosome interaction, highlighting MDV's critical role in cellular homeostasis regulation. Therefore, it is crucial to understand that MDV targeting the lysosomal pathway is significant. Recent research has revealed that soluble N‐ethylmaleimide‐sensitive factor attachment protein receptor (SNARE) facilitates the fusion of mitochondrial transport vesicles with lysosomes,^85^ which is essential for intracellular transport and degradation. SNARE proteins are conserved and mediate typical membrane fusion. They have been observed to form a four‐helix bundle via their coiled SNARE domain, promoting membrane fusion. Mitochondrial fusion is homotypic, SNARE‐independent, and regulated by outer mitochondrial membrane fusion proteins.^87^ Moreover, MDV‐lysosome fusion is heterotypic and SNARE involvement is critical for MDV targeting the lysosomal pathway.^88^ Syntaxin‐17 (STX17), a SNARE complex subset, detects mitochondrial outer membrane curvature via a unique clamping structure; its absence reduces MDV transport to lysosome fusion, impeding the process.^85^ Studies on yeast have revealed that the phosphorylated ubiquitin‐binding domain (P‐UB) interacts with SNARE, and STX17 directly binds P‐UB.^89^ Recent studies^79^ have shown that STX17, SNAP29, and VAMP7 form a ternary SNARE complex crucial for PINK1/Parkin‐dependent MDVs targeting lysosomes, affecting MDV formation and maturation. Familial early‐onset Parkinson's disease has been associated with biallelic PINK1 (PARK6) mutations and PARK2 gene functional loss, causing Parkin deficiency.^90^ Xanthine oxidase (XO) stimulates H_2_O_2_ production, thus inducing single‐membrane MDVs targeted towards lysosomes, rich in outer mitochondrial membrane proteins like TOM20 or VDAC.^45^, ^77^ Tollip plays a critical role in MDV transport and acts as an adaptor molecule in the endosomal system, interacting with ESCRT‐0 protein Tom1, facilitating vesicle maturation and cargo transport.^80^, ^83^ In addition, Tollip captures MDVs, aids their entry into the endosomal transport system, and recruits cytoplasmic Parkin, regulating TOM20+ MDV transport.^91^ Tollip and Parkin's interaction creates a sophisticated complex, with Rab7 adhering to mature MDVs, thereby directing them towards lysosomal degradation. Tollip and Rab7 are recycled within lysosomes for further functional utilisation, marking TOM20+ MDVs for degradation.^82^ VPS35, a retromer component, dissociates from the retromer, recognises TOM20‐carrying MDVs, and binds them with DRP1 oligomers^92^ to form TOM20+/DRP1+MDVs, which engulf DRP1 oligomers and transport them from the mitochondrial surface to the lysosome for breakdown.

### 3.2 MDV pathways targeting peroxisomes

The transportation pathway of MDVs to peroxisomes is a classic transport process;^89^ **^,^** ^93^ however, its mechanism remains elusive as the pathway is specific to lysosomes.^94^ A crucial player in this process is MAPL, a mitochondrial small ubiquitin‐related modifier (SUMO) E3 ligase that maintains the stability of DRP1, degrading mitofusin 2 (Mfn2), and facilitating mitochondrial fission.^76^ Several studies have demonstrated the widespread presence of MAPL in MDVs^89^ and its significance in the transport process. Moreover, the production of ROS via the XO/xanthine reaction can induce the formation of translocase of the outer membrane 20 (TOM20)‐positive MDVs, which selectively target MAPL to peroxisomes in specific cell types.^81^ The complex of VPS35 and VPS26 is essential for transporting MAPL+ MDVs to the peroxisome within the cell. This complex not only facilitates the organised transport of MDVs but also plays a key role in supporting cellular metabolism and various biological functions.^62^, ^95^ In addition, silencing VPS35 or VPS26 has been found to reduce the co‐localisation of MAPL+ MDVs with peroxisomes, which highlights the significance of these proteins in the targeting process.^94^ Although these findings highlight the possible targeting mechanism, further investigation is warranted to fully comprehend the complex processes involved in MDV's localisation to peroxisomes. The intricate interplay between the various proteins and cellular components indicates the complexity of MDV transport pathways and the need for comprehensive research to identify their precise mechanisms. Overall, the MDV pathways targeting peroxisomes comprise a series of intricate interactions involving MAPL‐, ROS‐, TOM20‐positive vesicles, VPS35, and VPS26. Although the insights gained from these findings are valuable in understanding the process, further research is still necessary to fully comprehend the specific mechanisms involved in the targeting of MDVs to peroxisomes to further advance the understanding of cellular homeostasis and identify novel approaches for therapeutic interventions aimed at targeting these crucial cellular pathways.

### 3.3 Other MDV transport pathways

MDVs, which accumulate on bacterial‐laden macrophage phagosomes,^96^ play a critical role in transporting SOD2 to phagolysosome to facilitate bacterial elimination.^83^ VPS proteins, such as VPS26 and VPS29, are integral to cargo sorting and transport to the TGN, thereby maintaining cellular homeostasis and efficient intracellular transport.^97^ During stress conditions, MVBs can serve as damage‐associated molecular patterns (DAMPs) to promote inflammation, or cells can package MDVs into EVs for release, potentially protecting against cellular damage. Todkar et al.^63^ investigated the pathway involving OPA1 and SNX9 in EVs and revealed that the mutations in Parkin and PINK1 are related to the loading of α‐synuclein into MDVs in EVs, which might be a cause of Parkinson's disease.^84^ MVBs with 200 to 1000 nm diameters contain numerous vesicles and can fuse with MDVs that carry TOM20.^13^

## 4 THE PHYSIOLOGICAL CONTRIBUTIONS OF MDVS

As mentioned above, MDVs are crucial for ensuring the balance and stability of the internal cellular environment. Its involvement in various physiological processes indicates its crucial role in the cell. Firstly, MDVs are involved upstream of mitochondrial autophagy and maintain mitochondrial homeostasis.^98^ Secondly, they can break down various dysfunctional mitochondrial proteins, which are crucial for cellular health and function.^99^ Furthermore, MDVs have an essential and multifunctional involvement in organelles communication, highlighting their multifaceted and crucial role within the cellular environment.^100^ Overall, these diverse functions highlight the significant impact of MDVs in the regulation of cellular function and maintenance.

### 4.1 MDVs act upstream of mitochondrial autophagy to maintain mitochondrial homeostasis

Mitophagy, a crucial mechanism for maintaining MQC,^101^ depends on the participation of PINK1 and Parkin, similar to MDVs.^102^ However, PINK1 or Parkin inhibition did not cause significant phenotypic alterations or neurodegeneration, indicating an alternate mechanism for mitochondrial engulfment and MDV formation in higher eukaryotes.^103^, ^104^ MDVs are released within a short frame of 2–6 h after exposure to mild stress, such as treatment with antimycin A, whereas mitophagy typically occurs between 12 and 24 h after the stress onset.^99^ This temporal distinction suggests that MDVs protect mitochondria from mitophagy, which is triggered by mitochondrial damage caused by the absence of PINK1 and Parkin.^31^ Furthermore, decreased MDVs have been associated with increased mitophagy, indicating the upstream regulatory role of MDVs in the maintenance of mitochondrial homeostasis. In addition, exposure of cells to lysosomal inhibitors such as bafilomycin A1 or pepstatin A/E‐64D can result in a substantial accumulation of MDVs within the cytoplasm as well as multivesicular bodies.^85^ Moreover, an in vitro budding study performed a quantitative assessment of the cargo released into multivesicular bodies and revealed an expulsion rate of about 4% of specific proteins every hour, thus highlighting the importance of MDVs in the steady‐state transport of cargo.^66^ **^,^** ^85^ McLelland et al. in 2016 indicated the pivotal role of MDVs in the intracellular trafficking of proteins.^85^ These findings provide valuable insights into the intricate mechanisms involved in the regulation of protein transport within the cell. During mild cellular stress, MDVs protect the structural integrity of mitochondria, potentially by impeding the progression of mitochondrial autophagy, thus serving as a pivotal upstream regulator of mitophagy.^32^ Moreover, during cellular homeostasis, the quantitative appraisal of MDV‐mediated cargo transportation significantly participates in maintaining cellular stability.^105^ MDVs act as upstream regulators of mitochondrial autophagy and are indispensable for the preservation of mitochondrial robustness and operational efficiency.^106^ Therefore, a comprehensive examination of the interplay between MDVs and mitochondrial autophagy is indispensable to understanding the processes that regulate MQC within the cellular milieu.^107^ Furthermore, it is crucial to understand theoretically and practically the causes of mitochondrial dysfunction in order to develop effective treatments for mitochondrial diseases.

### 4.2 Involvement of MDVs in the degradation of damaged mitochondrial proteins

A study^108^ found that when mitochondria are stimulated by oxidative stress. Oxidation‐induced damaged mitochondrial proteins can be encapsulated by MDV and then transported to lysosomes for degradation. Moreover, intracellular mitochondria can effectively germinate and form MDVs containing the mitochondrial oxidative proteins in the basal state or when cultured with galactose to increase aerobic metabolism. As previously stated, compared to mitochondrial autophagy, MDV, as the first line of defence, will be activated within a few minutes of mitochondrial stress, leading to a significant increase in production. Furthermore, before reaching the stress threshold and initiating mitochondrial autophagy for the clearance of damaged mitochondria, it is plausible for oxidised or impaired proteins in mitochondria to be transported through MDVs, alleviating mitochondrial damage.^19^ Thus, mitochondria can internally break down and remove damaged proteins, as well as promote mitophagy, which can facilitate the degradation of these proteins.^109^ **^,^** ^110^

### 4.3 The involvement of MDVs in inter‐organelle communication

MDVs have been found to mediate inter‐organelle communication.^111^ For example, to enhance its antimicrobial function, SOD2 shuttles as MDV cargo between mitochondria and phagosomes.^83^ The translocation of MAPL between the mitochondria and peroxisomes can promote the biogenesis of peroxisomes.^112^ The fusion of vesicles containing various peroxiproteins, proteins for peroxisome biogenesis, can stimulate the generation of immature peroxisomes. Furthermore, MDVs have also been implicated in the biogenesis of peroxisomes, which are essential for the different metabolic processes within cells. In addition, peroxisomal proteins PEX3 and PEX14 have been found to target the mitochondria and subsequently release MDVs, which then fuse with the vesicles derived from the ER containing PEX16, resulting in peroxisomal precursor structures.^113^ MDVs carrying the peroxisomal marker E3 ubiquitin ligase MAPL can also generate peroxisomes de novo or via the growth and division of the existing organelles. The literature has also reported the advantageous effects of the transfer of mitochondrial components via EVs.^114^ This translocation not only occurs between different organelles (such as the endoplasmic reticulum) but also extends across distant cells or tissues such as the oxidative mitochondrial components carried by EVs released from palmitate‐stressed adipocytes.^62^ This horizontal mitochondrial transfer could be employed as a preventive signal against heart muscle damage, indicating potential implications for regenerative medicine. Moreover, the injection of EVs obtained from energy‐stressed adipocytes in mice before coronary artery ligation has been observed to relieve damage caused by cardiac ischemia/reperfusion.^62^ These EVs have significant protective effects, as they are associated with improved cardiac function and decreased tissue damage.^61^ However, a study analysed Parkin knockout mice with reduced mitochondrial‐enriched EV production in adipocytes and revealed that this cardiac protective effect was absent, highlighting the importance of mitochondrial components in EV‐mediated cardioprotection.^115^ Researchers have also discovered that brown adipocytes when exposed to heat stress, release EVs containing oxidised mitochondrial components that can be internalised again by parental brown adipocytes. This process reduces peroxisome proliferator‐activated receptor gamma (PPAR‐γ) and uncoupling protein 1 (UCP1) protein levels in the recipient cells. These findings revealed the vital role of EVs in intercellular communication and the potential modulation of cellular metabolism and function. Some other studies have indicated similar mechanisms in the hearts of mice, where resident macrophages have been observed to engulf EVs that contain mitochondria released by cardiomyocytes.^116^ The tyrosine‐protein kinase Mer is a macrophage receptor, which is crucially involved in the phagocytic uptake of EVs by recognising phosphatidylserine residues on their surface.^117^ Thus, understanding these intercellular communication pathways is crucial for identifying new avenues for the development of targeted therapies for cardiovascular and metabolic diseases. In addition, mice subjected to catecholamine or coronary artery ligation stress have indicated increased engulfment of EVs containing mitochondria. This indicates the possible role of these EVs in stress response and tissue regeneration mechanisms, further validating the significance of intercellular mitochondrial transfer in maintaining cellular homeostasis and responding to physiological as well as pathological stressors. Moreover, EVs released by neural stem cells (NSCs) have been found to contain mitochondrial proteins and intact mitochondria with complete membrane potential and respiration ability.^107^ The addition of EVs to L929 Rho0 cells lacking mitochondrial DNA (mtDNA), successfully revived mitochondrial function and increased cell viability, highlighting the therapeutic promise of these EVs for addressing mitochondrial dysfunction and cellular damage.^118^ Overall, this intricate interaction between MDVs and EVs, which transport mitochondrial components, reveals the fascinating mechanisms by which cells maintain cellular homeostasis and respond to stress. These data indicate the possibilities for treatments and additional exploration in regenerative medicine, cardiovascular disease, and metabolic disorders (Figure 3).

## 5 INDUCTION OF IMMUNE RESPONSES BY MDVS

**FIGURE 4:**
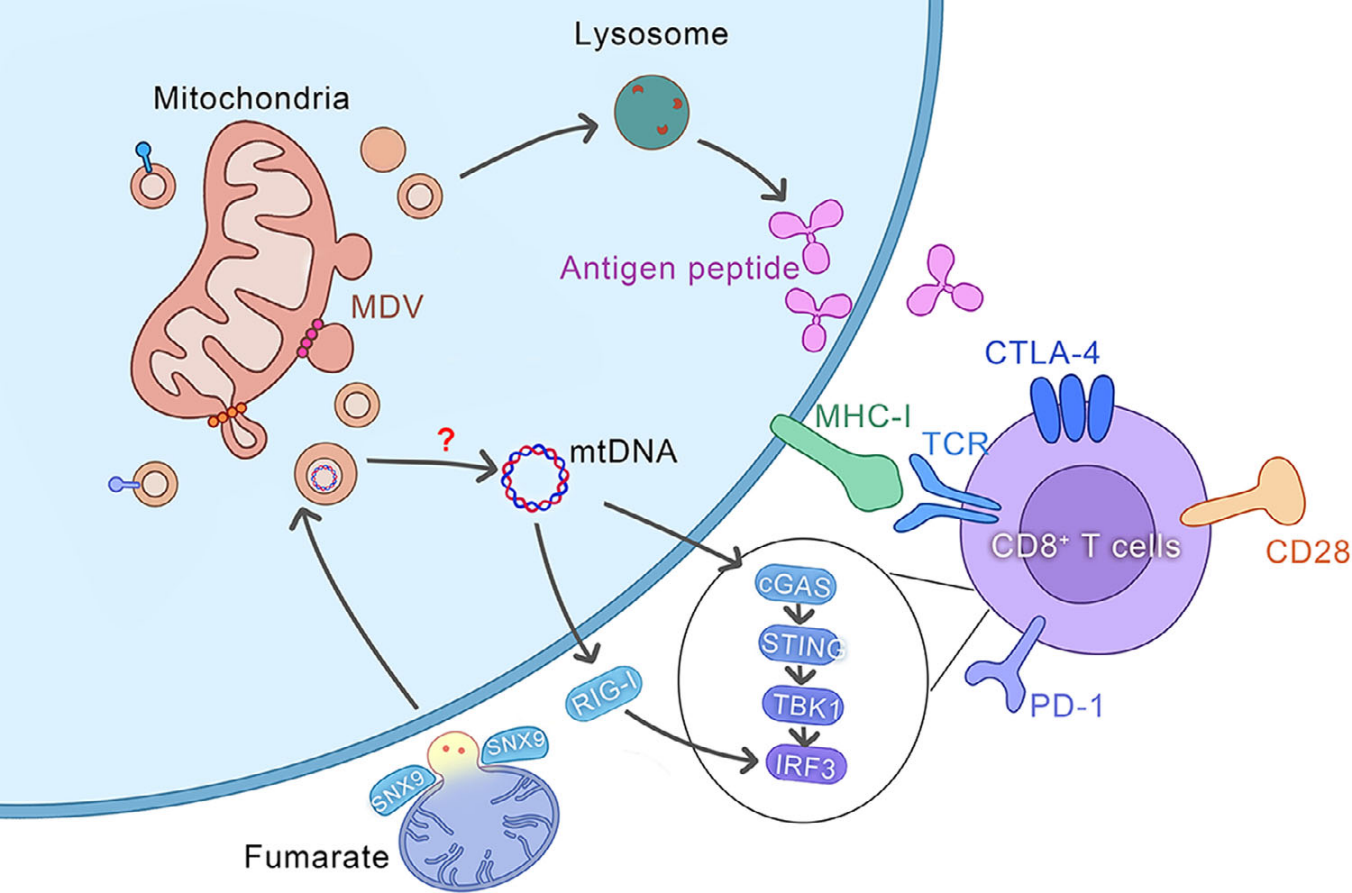
The mechanisms of mitochondrial‐derived vesicles (MDVs) involved in the mechanism of innate immune response. MDVs play a significant role in modulating innate immune responses. Through their transport of mitochondrial antigens to lysosomes, MDVs present these antigens to CD8+ T cells following processing by lysosomal hydrolases. This process results in the recognition of antigenic peptides on the cell surface, which triggers an immune response by the innate immune system. Another pathway through which MDVs contribute to innate immune responses involves perturbations in mitochondrial morphology and the release of mitochondrial DNA (mtDNA) into the cytoplasm due to fumarate accumulation. This release activates the cGAS–STING–TBK1 pathway, consequently initiating an innate immune response. These intricate mechanisms highlight the pivotal role of MDVs in modulating cellular immune signalling and innate immune defence.

In immune cells, MDVs serve as pivotal signals that trigger the body's immune response. This process comprises a series of finely regulated steps, beginning with the targeted recognition of MDVs, followed by their internalisation, subsequent release of components, and signal transduction, culminating in the activation of innate immune responses (Figure 4).

### 5.1 Phagocytosis of MDVs

For immune cells to process exogenous substances, it is crucial that MDVs undergo phagocytosis and internalisation. Upon MDV recognition by specific receptors on the immune cell surface, a complex series of phagocytic mechanisms are initiated to ensure their efficient internalisation into the cell interior.^62^ Phagocytosis is typically mediated by phagocytic receptors on the cell surface,^119^ which recognise and bind to specific molecules on the MDVs. Activation of these receptors stimulates dynamic alterations in the cell membrane, such as cytoskeleton remodelling, particularly of actin and tubulin.^120^ Rapid remodelling of the actin network enables the formation of pseudopodia.^121^ The formation of phagosomes is a highly dynamic process requiring the concerted action of various intracellular proteins, such as the Rho family of small GTPases.^122^ These proteins regulate the dynamics of the cytoskeleton to ensure the effective formation and movement of phagosomes into the cell. In phagosome formation, activation of the membrane fusion mechanism is a key step, which involves the interactions between SNARE proteins and members of the Rab GTPase superfamily.^123^ These proteins facilitate the fusion between the cell and phagosomal membranes, ensuring the effective release of MDV contents into the cell. The phagocytosis and internalisation of MDVs are complex processes involving the recognition by cell surface receptors, remodelling of the cytoskeleton, and activation of the membrane fusion mechanism. The synergistic action of these steps ensures the effective MDV internalisation by immune cells, providing a basis for further immune recognition and response

### 5.2 Recognition and signal transduction of MDV components

After MDV's internalisation, PRRs can effectively recognise the release of mitochondrial DNA, proteins, and other components within the cell.^123^ This process is crucial for immune cells to identify and respond to exogenous substances.Upon contact between antigen‐presenting dendritic cells (DCs) and T cells, T cells can deliver mitochondrial components (e.g., mtDNA) back to DCs via extracellular vesicles, triggering type I interferon responses and STING signalling in DCs.^124^ The cytosolic PRR cGAS can recognise and bind to exogenous or endogenous double‐stranded DNA^125^ and catalyses the production of the second messenger cGAMP, which then binds and activates the STING receptor. STING is a transmembrane protein predominantly located in the cytoplasm and can recognise cytosolic cyclic dinucleotides, such as cyclic GMP‐AMP (cGAMP).^126^ Once engulfed by DCs, the mtDNA in MDVs may be released into the cytoplasm, where it binds to cyclic GMP‐AMP synthase (cGAS) and catalyses cGAMP synthesis.^124^, ^127^ The interaction of cGAMP with the STING receptor triggers the activation of downstream signalling cascades, including the phosphorylation of TANK‐binding kinase 1 (TBK1) and interferon regulatory factor 3 (IRF3), activating type I interferon synthesis.^128^ Type I interferons are antiviral cytokines, which activate an antiviral state in immune cells, promoting their activation and migration.^129^ Other than the STING receptor, damaged MDVs may interact with immune cells via other receptors.^44^ For instance, Toll‐like receptors (TLRs), which are pattern recognition receptors (PRRs) expressed on the surface of immune cells, can recognise pathogen‐associated molecular patterns (PAMPs), such as bacterial lipopolysaccharides (LPS) or viral double‐stranded RNA (dsRNA).^130^ Furthermore, Absent in Melanoma 2 (AIM2), a cytosolic inflammasome receptor can recognise double‐stranded DNA and assemble it into an inflammasome.^131^ This process involves apoptosis‐associated speck‐like protein recruitment and caspase‐1 activation.^132^ **^,^** ^133^ Activated caspase‐1 cleaves the pro‐inflammatory cytokines pro‐IL‐1β and pro‐IL‐18, maturing and releasing them extracellularly to promote the development of inflammatory responses.^134^ Activation of these PRRs is not limited to the aforementioned mechanisms and they have been observed to be activated by other signalling pathways, such as the activation of interferon regulatory factors (IRFs) and nuclear factor‐κB (NF‐κB).^135^ IRFs are transcription factors that, when recognised for viral infection or intracellular DNA damage, induce the expression of type I interferons, activating an antiviral state in immune cells.^136^ NF‐κB, a key transcription factor for inflammatory responses, upon recognising inflammatory signals from both intracellular and extracellular sources can induce the expression of various inflammatory cytokines and chemokines, promoting the activation and migration of immune cells.^137^ The MDV components recognition and signal transduction is a complex process that involves the activation of multiple PRRs and the coordination of various signalling pathways.^138^ These mechanisms ensure that immune cells can effectively recognise and respond to signals carried by MDVs, thus maintaining immune homeostasis and defending against pathogen invasion.

### 5.3 MDV activates innate immune response

MDVs are involved in the transportation of mitochondrial antigens to lysosomes for antigen presentation, a process tightly regulated by numerous proteins. After undergoing degradation by lysosomal hydrolases, the antigenic peptides are transported to the cell surface, where they are identified by immune cells and elicit an immune response,^139^, ^140^, ^141^ triggering the innate immune response. Another pathway through which MDVs participate in the innate immune response involves early alterations in mitochondrial morphology and the release of mtDNA into the cytoplasm as a result of fumarate deficiency. This activates the cGAS–STING–TBK1 pathway, which stimulates the innate immune response in a manner partly reliant on RIG‐I. This phenotype is mediated by succinate and selectively occurs through MDVs that rely on SNX9.^34^ MDVs play a vital role in phagocytic cells by effectively eliminating bacterial pathogens by transporting superoxide dismutase 2 (SOD2). In the innate immune system, when phagocytic cells like macrophages or neutrophils experience stress, they generate ROS by triggering NADPH oxidase or stimulating mitochondrial metabolism. Mitochondrial ROS (mROS) can kill bacterial pathogens, thereby exerting its bactericidal effect.^91^ These findings indicate the significant role of MDVs in the immune response, as they are essential for antigen presentation, early activation of the innate immune response, and clearance of bacterial pathogens. Therefore, the elucidation of the mechanisms and pathways underlying MDV involvement in immune regulation is crucial for identifying novel strategies for developing targeted immunotherapies and offering profound insights into intercellular communication and immune system modulation. Investigation of MDVs and their role is crucial for increasing our understanding of immune responses and the development of innovative therapeutic interventions (Figure 4).

## 6 MDVS PROMOTE TUMOUR PROGRESSION

**FIGURE 5:**
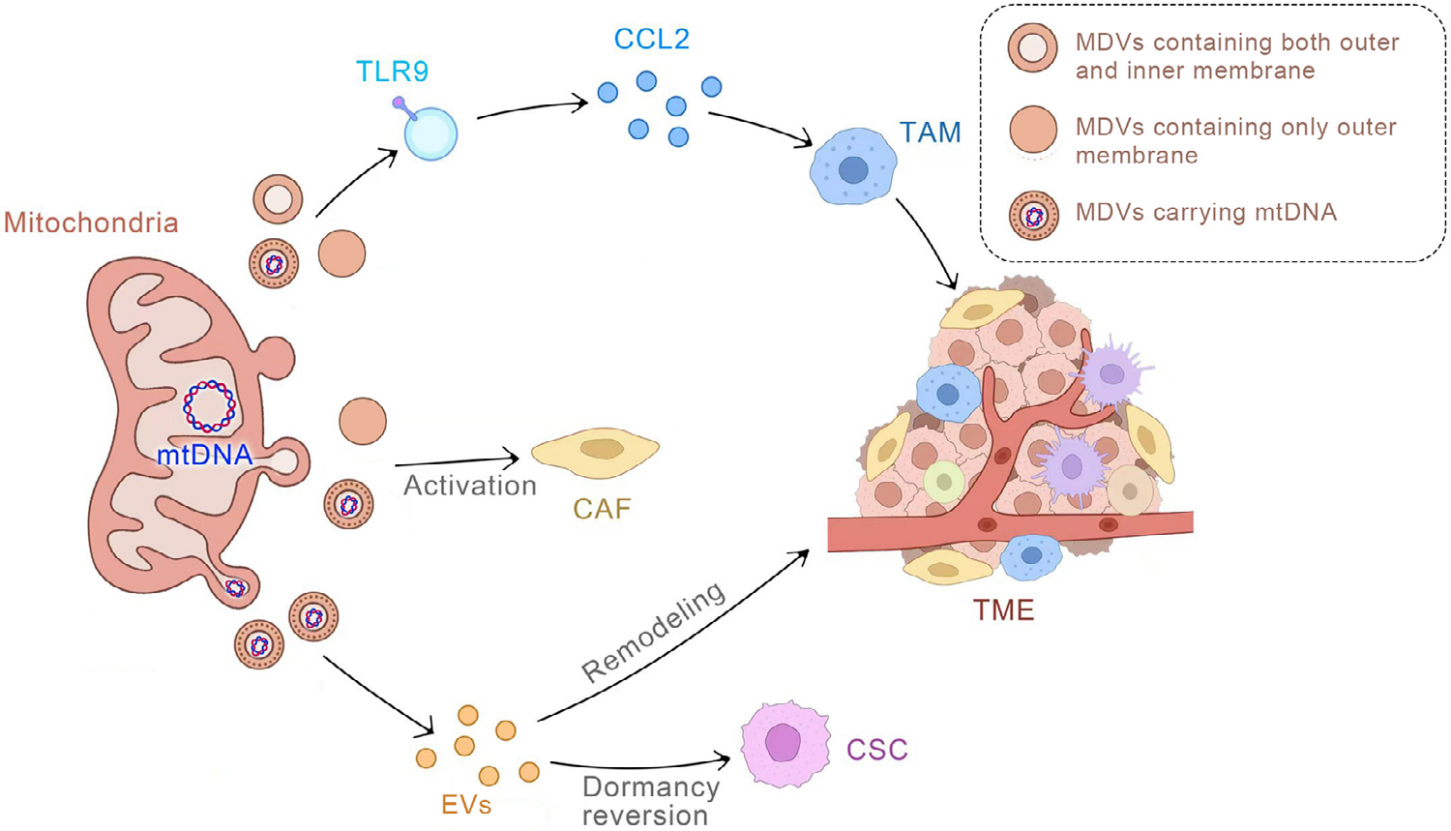
The mechanisms of mitochondria‐derived vesicles (MDVs) involved in tumour progression. Mitochondrial DNA (mtDNA) serves as a critical cargo within MDVs. Firstly, MDVs carrying mtDNA can activate fibroblasts, leading to the promotion of tumour progression. Secondly, MDVs carrying mtDNA can fuse with exosomes, thereby reshaping the tumour microenvironment. This process can also induce the dormancy reversal of cancer stem cells (CSCs) and facilitate tumour progression. Lastly, MDVs carrying mtDNA can activate Toll‐like receptor 9 (TLR9), resulting in the release of CCL2 which can impact tumour‐associated macrophages (TAMs) and strengthen the immunosuppression within the tumour microenvironment.

In cancer and other cell types with impaired mitochondrial engulfment, the generation of MDVs serves as a compensatory and adaptive mechanism to maintain mitochondrial health.^32^ **^,^** ^105^ **^,^** ^142^ When mitochondria cannot be effectively cleared through the engulfment process, MDVs are generated and then targeted for lysosomal clearance to enable the elimination of damaged mitochondrial particles. This process aids in preventing severe cellular consequences caused by imbalanced mitochondrial function by ensuring the elimination of dysfunctional mitochondrial fragments. MDVs can also transport mtDNA.^71^, ^143^ Dysfunctions in mtDNA, along with genetic mutations, are intricately associated with tumourigenesis and can contribute to varying degrees of tumour progression. For example, a prior study has reported that in hepatocellular carcinoma (HCC), aberrant mitochondrial fission mediated by DRP1 can effectively trigger a mtDNA stress response, leading to its release from mitochondria into the cytoplasm. This DRP1‐mediated mtDNA stress can then activate the TLR9 receptor signalling pathway and upregulate the secretion of chemokine CCL2. This in turn promotes the infiltration of tumour‐associated macrophages (TAMs) into the tumour microenvironment (TME), which causes immune tolerance of HCC tumour cells, thereby exacerbating HCC progression.^144^ Hence, MDVs carrying mutated mtDNA can promote tumour development through a complex mechanism that involves the MDV's interaction with the EVs pathway. It is well‐recognised that tumours are complex systems engaged in continuous communication with the microenvironment.Therefore, once targeted by MDV, the resulting MDV‐EVs play a crucial role in tumour development.^145^ Breast cancer cells release EVs rich in mtDNA that can enhance the expression of matrix metalloproteinase (MMP) and α5β1 integrin under glutamine deficiency conditions, promoting the invasiveness of recipient breast cancer cells.^35^ Acute myeloid leukaemia (AML) cells release increased levels of mitochondrial EVs during differentiation, and inhibiting the formation of these EVs can prevent myeloid cell differentiation.^146^ Melanoma cells release EVs rich in mtDNA that can induce cytokine production in macrophages, thereby inhibiting the cytotoxic T‐cell immune response in the tumour microenvironment.^125^ Moreover, EV‐mediated mitochondrial transfer may contribute to cancer drug resistance. In metastatic breast cancer patients with resistance to insulin therapy, circulating EVs rich in mtDNA were detected, which might act as a pro‐oncogenic signal to induce endocrine therapy resistance in breast cancer cells dependent on oxidative phosphorylation.^83^ EVs released by chemoresistant triple‐negative breast cancer cells can transfer functional mitochondria to sensitive triple‐negative breast cancer cells, leading to increased chemoresistance and tumourigenesis.^147^ Similarly, EVs from tumour‐activated stromal cells deliver mitochondria to malignant glioma cells, resulting in resistance to cancer radiotherapy and chemotherapy.^148^ In addition, in the plasma of patients with metastatic breast cancer, MDVs carrying mutated mtDNA have been detected. This mtDNA can be transferred by cancer‐associated fibroblasts through MDVs to restore oxidative phosphorylation in hormone therapy‐treated breast cancer cells^71^ (Figure 5).

## 7 POTENTIAL CLINICAL APPLICATIONS OF MDVS

The potential of MDV as a tumour therapy target primarily depends on two factors. Firstly, in clinical treatment, MDV can be employed as a vector to carry specific therapeutic agents, which can provide novel designs for immunotherapeutic strategies against tumours, and enhance treatment efficacy. Secondly, for diagnostics, MDVs are potential biomarkers for tumour diagnosis and prognosis assessment. By enabling earlier detection of tumours and evaluation of their progression, MDVs can assist physicians in providing more precise treatment regimens for patients.

### 7.1 The potential of MDV targeted therapy

As aforementioned, mtDNA serves as one of the cargoes transported by MDVs, effectively promoting tumour progression to some extent.^44^ This discovery not only highlights the significant role of mtDNA in tumour biology but also provides new avenues for developing novel cancer therapies. MDV‐targeted therapies against tumours are based on this phenomenon, which aims to modulate the function of mtDNA within MDVs using mitochondria‐targeted molecules. Key methods include the application of nanocarrier systems to deliver drugs specifically targeting mtDNA. Advancements in nanotechnology have offered new possibilities for drug delivery. For instance, Hall et al. utilised delocalised lipophilic cations to prepare mitochondria‐targeting agents, designing molecular‐based nanocarriers that selectively accumulate in mitochondria under a high transmembrane potential (ΔΨm) and deliver bioactive substances.^149^ This approach enhances drug targeting and efficacy while reducing normal cell toxicity by employing metal complexes for targeted delivery against mtDNA. It has been indicated that metal elements are significantly associated with biochemical reactions. Certain metals can participate in mitochondrial reactions, while others selectively bind to DNA and stabilise complexes. Jiang et al.^150^ developed a nanocatalytic drug, MSN‐Ru^2+^/Fe^2+^, composed of mesoporous silica nanoparticles carrying Fe^2+^‐Ru^2+^ complexes. Ru^2+^ complexes bind to mtDNA, and Fe^2+^ Fenton reagents promote ROS production in mitochondria through Fenton reactions, inducing apoptosis. Phototherapy is employed to activate specific drugs or substances targeting mtDNA to promote cancer cell destruction. It uses specific light wavelengths to activate drugs or substances for therapeutic purposes. Yang et al.^151^ precisely assembled biotinylated Pt (IV) prodrug derivatives with IR780 at a 1:1 molecular ratio, achieving mitochondria‐targeted chemophotothermal therapy in A549R tumour cells under near‐infrared (NIR) irradiation, nearly eliminating tumours. This method has combined advantages of chemotherapy and photothermal therapy and therefore, can precisely target tumour cells with minimal damage to surrounding normal tissues. In summary, the rapid development of mtDNA‐targeted therapies suggests that MDVs can serve as potential carriers of mtDNA and have a significant potential as a target for cancer treatment. These innovative targeted strategies not only enhance therapeutic efficacy but also reduce side effects, offering new hope for cancer patients. Future advancements based on the comprehensive knowledge of the biological role of mtDNA and MDVs, as well as further development of nanotechnology and phototherapy, will yield more efficient and safer cancer treatments.

### 7.2 Potential of MDVs as biomarkers in tumour diagnosis and prognosis

MDVs hold significant clinical potential in tumour‐targeted therapies, extending beyond therapeutic applications to encompass crucial diagnostic implications. As potential biomarkers, the detection of MDVs in blood offers a novel approach to assessing the vitality of mitochondria within the cardiovascular system, underscoring their pivotal role in diagnosis.^152^ It has been observed that circulating MDVs serve as indicators of mitochondrial dysfunction.^153^ Therefore, MDVs have the potential for diagnostic purposes and can serve as innovative biomarkers in the medical field to evaluate mitochondrial function and related diseases. By assessing the distribution and characteristics of MDVs across various tumours, it is possible to uncover their potential as biomarkers for diagnosing and assessing a range of diseases. This could pave the way for personalised treatments and novel clinical diagnostic strategies. The anticipated outcomes of this prospective research are poised to significantly advance the medical field, offering more comprehensive and precise support for existing therapeutic and diagnostic approache.^154^ Altogether, the potential applications of MDVs in diagnosis merit further and in‐depth investigation, which can help provide valuable insights into the medical field and open new diagnostic and therapeutic avenues and methodologies in the future.^155^ It is imperative to acknowledge and enhance these potential applications through extensive research and collaborative efforts, as they are crucial for revolutionising and enhancing clinical diagnostic and therapeutic paradigms.

## 8 CONCLUSION AND FUTURE PERSPECTIVES

In summary, MDVs have become a focal point in cancer research due to their role in tumour progression and interaction with the TME. These vesicles carry bioactive molecules that modulate tumour cell behaviour and immune responses, making them potential therapeutic targets. Furthermore, they are being investigated as biomarkers are being explored for early diagnosis and prognosis assessment. Understanding how MDVs influence the tumour immune microenvironment could lead to novel targeted therapies, improving the efficacy of cancer treatments and offering new avenues for early diagnosis and personalised care. Future studies will aim to elucidate its biological functions and regulatory networks to develop more effective and safe cancer treatments.

## AUTHOR CONTRIBUTIONS

XP, YG and JL conducted the research study and drafted the manuscript. XS and WL provided assistance during the revision and drafting process. YM, XL and HL contributed to the conceptual framework, supervised the study, and revised the manuscript. All authors carefully reviewed the final manuscript and approved it for publication.

## CONFLICT OF INTEREST STATEMENT

The authors declare that they have no competing interests.

## References

[1] Jasra IT , Cuesta‐Gomez N , Verhoeff K , Marfil‐Garza BA , Dadheech N , Shapiro AMJ . Mitochondrial regulation in human pluripotent stem cells during reprogramming and beta cell differentiation. Front Endocrinol (Lausanne). 2023;14:1236472. 37929027 10.3389/fendo.2023.1236472 PMC10623316

[2] Rongvaux A . Innate immunity and tolerance toward mitochondria. Mitochondrion. 2018;41:14‐20. 29054471 10.1016/j.mito.2017.10.007

[3] Zhang SS , Zhou S , Crowley‐McHattan ZJ , Wang RY , Li JP . A review of the role of endo/sarcoplasmic reticulum‐mitochondria Ca(2+) transport in diseases and skeletal muscle function. Int J Environ Res Public Health. 2021;18. 10.3390/ijerph18083874 PMC8067840 33917091

[4] Amador‐Martínez I , Aparicio‐Trejo OE , Bernabe‐Yepes B , et al. Mitochondrial impairment: a link for inflammatory responses activation in the cardiorenal syndrome Type 4. Int J Mol Sci. 2023;24. 10.3390/ijms242115875 PMC10650149 37958859

[5] Barabino S , Lombardi S , Zilocchi M . Keep in touch: a perspective on the mitochondrial social network and its implication in health and disease. Cell Death Discov. 2023;9:417. 37973903 10.1038/s41420-023-01710-9 PMC10654391

[6] Campello S , Strappazzon F , Cecconi F . Mitochondrial dismissal in mammals, from protein degradation to mitophagy. Biochim Biophys Acta. 2014;1837:451‐460. 24275087 10.1016/j.bbabio.2013.11.010

[7] Mukkala AN , Jerkic M , Khan Z , Szaszi K , Kapus A , Rotstein O . Therapeutic effects of mesenchymal stromal cells require mitochondrial transfer and quality control. Int J Mol Sci. 2023;24. 10.3390/ijms242115788 PMC10647450 37958771

[8] Li Y , Fan J , Zhu W , Niu Y , Zhang A . Therapeutic potential targeting podocyte mitochondrial dysfunction in focal segmental glomerulosclerosis. Kidney Dis (Basel). 2023;9:254‐264. 37900001 10.1159/000530344 PMC10601935

[9] Zhou Y , Suo W , Zhang X , et al. Targeting mitochondrial quality control for diabetic cardiomyopathy: therapeutic potential of hypoglycemic drugs. Biomed Pharmacother. 2023;168:115669. 37820568 10.1016/j.biopha.2023.115669

[10] Ma J , Liu L , Song L , et al. Integration of FUNDC1‐associated mitochondrial protein import and mitochondrial quality control contributes to TDP‐43 degradation. Cell Death Dis. 2023;14:735. 37951930 10.1038/s41419-023-06261-6 PMC10640645

[11] Liu Y , Fu T , Li G , et al. Mitochondrial transfer between cell crosstalk – an emerging role in mitochondrial quality control. Age Res Rev. 2023;91:102038. 10.1016/j.arr.2023.102038 37625463

[12] Gorbunova AS , Zamaraev AV , Yapryntseva MA , et al. Prognostic signature based on mitochondria quality control proteins for the prediction of lung adenocarcinoma patients survival. Cell Death Discov. 2023;9:352. 37749074 10.1038/s41420-023-01649-x PMC10519931

[13] Soubannier V , McLelland G , Zunino R , et al. A vesicular transport pathway shuttles cargo from mitochondria to lysosomes. Curr Biol. 2012;22:135‐141. 22226745 10.1016/j.cub.2011.11.057

[14] An X , Ma X , Liu H , et al. Inhibition of PDGFRbeta alleviates endothelial cell apoptotic injury caused by DRP‐1 overexpression and mitochondria fusion failure after mitophagy. Cell Death Dis. 2023;14:756. 37980402 10.1038/s41419-023-06272-3 PMC10657461

[15] Noone J , Rochfort KD , O'Sullivan F , O'Gorman DJ . SIRT4 is a regulator of human skeletal muscle fatty acid metabolism influencing inner and outer mitochondrial membrane‐mediated fusion. Cell Signal. 2023;112:110931. 37858614 10.1016/j.cellsig.2023.110931

[16] Lee H , Smith SB , Sheu SS , Yoon Y . The short variant of optic atrophy 1 (OPA1) improves cell survival under oxidative stress. J Biol Chem. 2020;295:6543‐6560. 32245890 10.1074/jbc.RA119.010983 PMC7212625

[17] Chen L , Qin Y , Liu B , et al. PGC‐1alpha‐mediated mitochondrial quality control: molecular mechanisms and implications for heart failure. Front Cell Dev Biol. 2022;10:871357. 35721484 10.3389/fcell.2022.871357 PMC9199988

[18] Yoshii SR , Mizushima N . Autophagy machinery in the context of mammalian mitophagy. Biochim Biophys Acta. 2015;1853:2797‐2801. 25634658 10.1016/j.bbamcr.2015.01.013

[19] Bozi LH , Bechara LR , Dos Santos AF , Campos JC . Mitochondrial‐derived vesicles: a new player in cardiac mitochondrial quality control. J Physiol. 2016;594:6077‐6078. 27800623 10.1113/JP273124 PMC5088229

[20] Borcherding N , Brestoff JR . The power and potential of mitochondria transfer. Nature. 2023;623:283‐291. 37938702 10.1038/s41586-023-06537-z PMC11590279

[21] Saha T , Dash C , Jayabalan R , et al. Intercellular nanotubes mediate mitochondrial trafficking between cancer and immune cells. Nat Nanotechnol. 2022;17:98‐106. 34795441 10.1038/s41565-021-01000-4 PMC10071558

[22] Li S , Sheng ZH . Energy matters: presynaptic metabolism and the maintenance of synaptic transmission. Nat Rev Neurosci. 2022;23:4‐22. 34782781 10.1038/s41583-021-00535-8

[23] Kubat GB , Bouhamida E , Ulger O , et al. Mitochondrial dysfunction and skeletal muscle atrophy: causes, mechanisms, and treatment strategies. Mitochondrion. 2023;72:33‐58. 37451353 10.1016/j.mito.2023.07.003

[24] Siegel RL , Giaquinto AN , Jemal A . Cancer statistics, 2024. CA Cancer J Clin. 2024;74:12‐49. 38230766 10.3322/caac.21820

[25] Ma S , Zhang J , Yan T , et al. Novel strategies to reverse chemoresistance in colorectal cancer. Cancer Med. 2023;12:11073‐11096. 36645225 10.1002/cam4.5594 PMC10242875

[26] Zhou R , Tang X , Wang Y . Emerging strategies to investigate the biology of early cancer. Nat Rev Cancer. 2024;24:850‐866. 39433978 10.1038/s41568-024-00754-y

[27] Liu Z , Chen J , Ren Y , et al. Multi‐stage mechanisms of tumor metastasis and therapeutic strategies. Signal Transduct Targeted Ther. 2024;9:270. 10.1038/s41392-024-01955-5 PMC11467208 39389953

[28] Esposito M , Ganesan S , Kang Y . Emerging strategies for treating metastasis. Nat Cancer. 2021;2:258‐270. 33899000 10.1038/s43018-021-00181-0 PMC8064405

[29] Jokhadze N , Das A , Dizon DS . Global cancer statistics: a healthy population relies on population health. CA Cancer J Clin. 2024;74:224‐226. 38572764 10.3322/caac.21838

[30] Watson DC , Bayik D , Storevik S , et al. GAP43‐dependent mitochondria transfer from astrocytes enhances glioblastoma tumorigenicity. Nat Cancer. 2023;4:648‐664. 37169842 10.1038/s43018-023-00556-5 PMC10212766

[31] Sugiura A , McLelland GL , Fon EA , McBride HM . A new pathway for mitochondrial quality control: mitochondrial‐derived vesicles. EMBO J. 2014;33:2142‐2156. 25107473 10.15252/embj.201488104 PMC4282503

[32] Towers CG , Wodetzki DK , Thorburn J , Smith KR , Caino MC , Thorburn A . Mitochondrial‐derived vesicles compensate for loss of LC3‐mediated mitophagy. Dev Cell. 2021;56:2029‐2042 e2025. 34171288 10.1016/j.devcel.2021.06.003 PMC8319140

[33] Gagliardi S , Mitruccio M , Di Corato R , et al. Defects of mitochondria‐lysosomes communication induce secretion of mitochondria‐derived vesicles and drive chemoresistance in ovarian cancer cells. Cell Commun Signal. 2024;22:165. 38448982 10.1186/s12964-024-01507-y PMC10916030

[34] Zecchini V , Paupe V , Herranz‐Montoya I , et al. Fumarate induces vesicular release of mtDNA to drive innate immunity. Nature. 2023;615:499‐506. 36890229 10.1038/s41586-023-05770-w PMC10017517

[35] Rabas N , Palmer S , Mitchell L , et al. PINK1 drives production of mtDNA‐containing extracellular vesicles to promote invasiveness. J Cell Biol. 2021;220. 10.1083/jcb.202006049 PMC8641410 34623384

[36] Neuspiel M , Schauss AC , Braschi E , et al. Cargo‐selected transport from the mitochondria to peroxisomes is mediated by vesicular carriers. Curr Biol. 2008;18:102‐108. 18207745 10.1016/j.cub.2007.12.038

[37] Yamashita A , Fujimoto M , Katayama K , Yamaoka S , Tsutsumi N , Arimura S . Formation of mitochondrial outer membrane derived protrusions and vesicles in *Arabidopsis thaliana* . PLoS One. 2016;11:e0146717. 26752045 10.1371/journal.pone.0146717 PMC4713473

[38] Ramirez A , Old W , Selwood DL , Liu X . Cannabidiol activates PINK1‐Parkin‐dependent mitophagy and mitochondrial‐derived vesicles. Eur J Cell Biol. 2022;101:151185. 34915361 10.1016/j.ejcb.2021.151185 PMC8816654

[39] Schubert AF , Gladkova C , Pardon E , et al. Structure of PINK1 in complex with its substrate ubiquitin. Nature. 2017;552:51‐56. 29160309 10.1038/nature24645 PMC6020998

[40] McLelland GL , Soubannier V , Chen CX , McBride HM , Fon EA . Parkin and PINK1 function in a vesicular trafficking pathway regulating mitochondrial quality control. EMBO J. 2014;33:282‐295. 24446486 10.1002/embj.201385902 PMC3989637

[41] Matheoud D , Sugiura A , Bellemare‐Pelletier A , et al. Parkinson's disease‐related proteins PINK1 and Parkin repress mitochondrial antigen presentation. Cell. 2016;166:314‐327. 27345367 10.1016/j.cell.2016.05.039

[42] Popov LD . Mitochondrial‐derived vesicles: recent insights. J Cell Mol Med. 2022;26:3323‐3328. 35582908 10.1111/jcmm.17391 PMC9189329

[43] Schöneberg J , Lehmann M , Ullrich A , et al. Lipid‐mediated PX‐BAR domain recruitment couples local membrane constriction to endocytic vesicle fission. Nat Commun. 2017;8:15873. 28627515 10.1038/ncomms15873 PMC5481832

[44] König T , McBride HM . Mitochondrial‐derived vesicles in metabolism, disease, and aging. Cell Metab. 2024;36:21‐35. 38171335 10.1016/j.cmet.2023.11.014

[45] König T , Nolte H , Aaltonen MJ , et al. MIROs and DRP1 drive mitochondrial‐derived vesicle biogenesis and promote quality control. Nat Cell Biol. 2021;23:1271‐1286. 34873283 10.1038/s41556-021-00798-4

[46] Picca A , Guerra F , Calvani R , et al. Generation and release of mitochondrial‐derived vesicles in health, aging and disease. J Clin Med. 2020;9. 10.3390/jcm9051440 PMC7290979 32408624

[47] Chaudhary PK , Kim S , Kim S . Shedding light on the cell biology of platelet‐derived extracellular vesicles and their biomedical applications. Life (Basel). 2023;13. 10.3390/life13061403 PMC10326820 37374185

[48] Pegtel DM , Gould SJ . Exosomes. Annu Rev Biochem. 2019;88:487‐514. 31220978 10.1146/annurev-biochem-013118-111902

[49] Yang S , Zheng B , Raza F , et al. Tumor‐derived microvesicles for cancer therapy. Biomater Sci. 2024;12:1131‐1150. 38284828 10.1039/d3bm01980b

[50] Gao P , Zhou L , Wu J , et al. Riding apoptotic bodies for cell‐cell transmission by African swine fever virus. Proc Natl Acad Sci USA. 2023;120:e2309506120. 37983498 10.1073/pnas.2309506120 PMC10691326

[51] Silva AM , Lázaro‐Ibáñez E , Gunnarsson A , et al. Quantification of protein cargo loading into engineered extracellular vesicles at single‐vesicle and single‐molecule resolution. J Extracellul Vesic. 2021;10:e12130. 10.1002/jev2.12130 PMC8329990 34377376

[52] Miranda KC , Bond DT , McKee M , et al. Nucleic acids within urinary exosomes/microvesicles are potential biomarkers for renal disease. Kidney Int. 2010;78:191‐199. 20428099 10.1038/ki.2010.106 PMC4451567

[53] Kowal J , Arras G , Colombo M , et al. Proteomic comparison defines novel markers to characterize heterogeneous populations of extracellular vesicle subtypes. Proc Natl Acad Sci USA. 2016;113:E968‐977. 26858453 10.1073/pnas.1521230113 PMC4776515

[54] Isaac R , Reis FCG , Ying W , Olefsky JM . Exosomes as mediators of intercellular crosstalk in metabolism. Cell Metab. 2021;33:1744‐1762. 34496230 10.1016/j.cmet.2021.08.006 PMC8428804

[55] Corbeil D , Santos MF , Karbanová J , Kurth T , Rappa G , Lorico A . Uptake and fate of extracellular membrane vesicles: nucleoplasmic reticulum‐associated late endosomes as a new gate to intercellular communication. Cells. 2020;9. 10.3390/cells9091931 PMC7563309 32825578

[56] Lark DS , Stemmer K , Ying W , Crewe C . A brief guide to studying extracellular vesicle function in the context of metabolism. Nat Metab. 2024;6:1839‐1841. 39187615 10.1038/s42255-024-01112-w

[57] Théry C , Witwer KW , Aikawa E , et al. Minimal information for studies of extracellular vesicles 2018 (MISEV2018): a position statement of the International Society for Extracellular Vesicles and update of the MISEV2014 guidelines. J Extracellul Vesic. 2018;7:1535750. 10.1080/20013078.2018.1535750 PMC6322352 30637094

[58] Xiang H , Bao C , Chen Q , et al. Extracellular vesicles (EVs)' journey in recipient cells: from recognition to cargo release. J Zhejiang Univ Sci B. 2024;25:633‐655. 39155778 10.1631/jzus.B2300566 PMC11337091

[59] Ryan TA , Phillips EO , Collier CL , et al. Tollip coordinates Parkin‐dependent trafficking of mitochondrial‐derived vesicles. EMBO J. 2020;39:e102539. 32311122 10.15252/embj.2019102539 PMC7265236

[60] Zhou X , Liu S , Lu Y , Wan M , Cheng J , Liu J . MitoEVs: a new player in multiple disease pathology and treatment. J Extracellul Vesic. 2023;12:e12320. 10.1002/jev2.12320 PMC10065981 37002588

[61] Crewe C , Funcke J , Li S , et al. Extracellular vesicle‐based interorgan transport of mitochondria from energetically stressed adipocytes. Cell Metab. 2021;33:1853‐1868 e1811. 34418352 10.1016/j.cmet.2021.08.002 PMC8429176

[62] Rosina M , Ceci V , Turchi R , et al. Ejection of damaged mitochondria and their removal by macrophages ensure efficient thermogenesis in brown adipose tissue. Cell Metab. 2022;34:533‐548 e512. 35305295 10.1016/j.cmet.2022.02.016 PMC9039922

[63] Todkar K , Chikhi L , Desjardins V , El‐Mortada F , Pépin G , Germain M . Selective packaging of mitochondrial proteins into extracellular vesicles prevents the release of mitochondrial DAMPs. Nat Commun. 2021;12:1971. 33785738 10.1038/s41467-021-21984-w PMC8009912

[64] Vikramdeo KS , Anand S , Sudan SK , et al. Profiling mitochondrial DNA mutations in tumors and circulating extracellular vesicles of triple‐negative breast cancer patients for potential biomarker development. FASEB Bioadv. 2023;5:412‐426. 37810173 10.1096/fba.2023-00070 PMC10551276

[65] Liang W , Sagar S , Ravindran R , et al. Mitochondria are secreted in extracellular vesicles when lysosomal function is impaired. Nat Commun. 2023;14:5031. 37596294 10.1038/s41467-023-40680-5 PMC10439183

[66] Soubannier V , Rippstein P , Kaufman BA , Shoubridge EA , McBride HM . Reconstitution of mitochondria derived vesicle formation demonstrates selective enrichment of oxidized cargo. PLoS One. 2012;7:e52830. 23300790 10.1371/journal.pone.0052830 PMC3530470

[67] Han Q , Li W , Hu K , et al. Exosome biogenesis: machinery, regulation, and therapeutic implications in cancer. Mol Cancer. 2022;21:207. 36320056 10.1186/s12943-022-01671-0 PMC9623991

[68] Heyn J , Heuschkel MA , Goettsch C . Mitochondrial‐derived vesicles‐link to extracellular vesicles and implications in cardiovascular disease. Int J Mol Sci. 2023;24. 10.3390/ijms24032637 PMC9917113 36768960

[69] Kalkavan H , Chen MJ , Crawford JC , et al. Sublethal cytochrome c release generates drug‐tolerant persister cells. Cell. 2022;185:3356‐3374 e3322. 36055199 10.1016/j.cell.2022.07.025 PMC9450215

[70] Schwager SC , Reinhart‐King CA . Mechanobiology of microvesicle release, uptake, and microvesicle‐mediated activation. Curr Top Membr. 2020;86:255‐278. 33837695 10.1016/bs.ctm.2020.08.004

[71] Sansone P , Savini C , Kurelac I , et al. Packaging and transfer of mitochondrial DNA via exosomes regulate escape from dormancy in hormonal therapy‐resistant breast cancer. Proc Natl Acad Sci USA. 2017;114:E9066‐E9075. 29073103 10.1073/pnas.1704862114 PMC5664494

[72] Kalluri R , LeBleu VS . The biology, function, and biomedical applications of exosomes. Science (New York, NY). 2020;367. 10.1126/science.aau6977 PMC7717626 32029601

[73] Tickner JA , Richard DJ , O'Byrne KJ . EV, microvesicles/microRNAs and stem cells in cancer. Adv Exp Med Biol. 2018;1056:123‐135. 29754178 10.1007/978-3-319-74470-4_8

[74] Meldolesi J . Exosomes and ectosomes in intercellular communication. Curr Biol. 2018;28:R435‐R444. 29689228 10.1016/j.cub.2018.01.059

[75] Zhang M , Schekman R . Cell biology. Unconventional secretion, unconventional solutions. Science (New York, NY). 2013;340:559‐561. 10.1126/science.1234740 23641104

[76] Cadete VJJ , Deschênes S , Cuillerier A , et al. Formation of mitochondrial‐derived vesicles is an active and physiologically relevant mitochondrial quality control process in the cardiac system. J Physiol. 2016;594:5343‐5362. 27311616 10.1113/JP272703 PMC5023710

[77] Fan K , Li Y , Wang H , et al. Stress‐induced metabolic disorder in peripheral CD4(+) T cells leads to anxiety‐like behavior. Cell. 2019;179:864‐879 e819. 31675497 10.1016/j.cell.2019.10.001

[78] Juhász G . A mitochondrial‐derived vesicle HOPS to endolysosomes using Syntaxin‐17. J Cell Biol. 2016;214:241‐243. 27458131 10.1083/jcb.201607024 PMC4970333

[79] Hao T , Yu J , Wu Z , et al. Hypoxia‐reprogramed megamitochondrion contacts and engulfs lysosome to mediate mitochondrial self‐digestion. Nat Commun. 2023;14:4105. 37433770 10.1038/s41467-023-39811-9 PMC10336010

[80] Hayashi Y , Takatori S , Warsame WY , Tomita T , Fujisawa T , Ichijo H . TOLLIP acts as a cargo adaptor to promote lysosomal degradation of aberrant ER membrane proteins. EMBO J. 2023;42:e114272. 37929762 10.15252/embj.2023114272 PMC10690474

[81] Ponzetti M , Ucci A , Puri C , et al. Effects of osteoblast‐derived extracellular vesicles on aggressiveness, redox status and mitochondrial bioenergetics of MNNG/HOS osteosarcoma cells. Front Oncol. 2022;12:983254. 36544705 10.3389/fonc.2022.983254 PMC9762506

[82] Wang W , Wang X , Fujioka H , et al. Parkinson's disease‐associated mutant VPS35 causes mitochondrial dysfunction by recycling DLP1 complexes. Nat Med. 2016;22:54‐63. 26618722 10.1038/nm.3983 PMC4826611

[83] Abuaita BH , Schultz TL , O'Riordan MX . Mitochondria‐derived vesicles deliver antimicrobial reactive oxygen species to control phagosome‐localized *Staphylococcus aureus* . Cell Host Microbe. 2018;24:625‐636 e625. 30449314 10.1016/j.chom.2018.10.005 PMC7323595

[84] Herman S , Djaldetti R , Mollenhauer B , Offen D . CSF‐derived extracellular vesicles from patients with Parkinson's disease induce symptoms and pathology. Brain. 2023;146:209‐224. 35881523 10.1093/brain/awac261

[85] McLelland GL , Lee SA , McBride HM , Fon EA . Syntaxin‐17 delivers PINK1/parkin‐dependent mitochondrial vesicles to the endolysosomal system. J Cell Biol. 2016;214:275‐291. 27458136 10.1083/jcb.201603105 PMC4970327

[86] Wurm CA , Neumann D , Lauterbach MA , et al. Nanoscale distribution of mitochondrial import receptor Tom20 is adjusted to cellular conditions and exhibits an inner‐cellular gradient. Proc Natl Acad Sci USA. 2011;108:13546‐13551. 21799113 10.1073/pnas.1107553108 PMC3158204

[87] Gao S , Hu J . mitochondrial fusion: the machineries in and out. Trends Cell Biol. 2021;31:62‐74. 33092941 10.1016/j.tcb.2020.09.008

[88] Picca A , Guerra F , Calvani R , et al. Mitochondrial dysfunction and aging: insights from the analysis of extracellular vesicles. Int J Mol Sci. 2019;20. 10.3390/ijms20040805 PMC6412692 30781825

[89] Kim P . Peroxisome biogenesis: a union between two organelles. Curr Biol. 2017;27:R271‐R274. 28376335 10.1016/j.cub.2017.02.052

[90] Gaweda‐Walerych K , Sitek EJ , Narożańska E , Buratti E . Parkin beyond Parkinson's disease – a functional meaning of Parkin downregulation in TDP‐43 proteinopathies. Cells. 2021;10. 10.3390/cells10123389 PMC8699658 34943897

[91] Ryan TA , Tumbarello DA . A central role for mitochondrial‐derived vesicles in the innate immune response: implications for Parkinson's disease. Neural Regen Res. 2021;16:1779‐1780. 33510074 10.4103/1673-5374.306074 PMC8328781

[92] Zhou Q , Qi F , Zhou C , et al. VPS35 promotes gastric cancer progression through integrin/FAK/SRC signalling‐mediated IL‐6/STAT3 pathway activation in a YAP‐dependent manner. Oncogene. 2024;43:106‐122. 37950040 10.1038/s41388-023-02885-2 PMC10774127

[93] Roberts RF , Bayne AN , Goiran T , et al. Proteomic profiling of mitochondrial‐derived vesicles in brain reveals enrichment of respiratory complex sub‐assemblies and small TIM chaperones. J Proteome Res. 2021;20:506‐517. 33242952 10.1021/acs.jproteome.0c00506

[94] Mohanty A , Zunino R , Soubannier V , Dilipkumar S . A new functional role of mitochondria‐anchored protein ligase in peroxisome morphology in mammalian cells. J Cell Biochem. 2021;122:1686‐1700. 34322908 10.1002/jcb.30114

[95] Lu Y , He P , Zhang Y , Ren Y , Zhang L . The emerging roles of retromer and sorting nexins in the life cycle of viruses. Virol Sin. 2022;37:321‐330. 35513271 10.1016/j.virs.2022.04.014 PMC9057928

[96] Wang L , Klionsky DJ , Shen HM . The emerging mechanisms and functions of microautophagy. Nat Rev Mol Cell Biol. 2023;24:186‐203. 36097284 10.1038/s41580-022-00529-z

[97] Hernandez DG , Reed X , Singleton AB . Genetics in Parkinson disease: mendelian versus non‐Mendelian inheritance. J Neurochem. 2016;139(Suppl 1):59‐74. 27090875 10.1111/jnc.13593 PMC5155439

[98] Jiao H , Jiang D , Hu X , et al. Mitocytosis, a migrasome‐mediated mitochondrial quality‐control process. Cell. 2021;184:2896‐2910 e2813. 34048705 10.1016/j.cell.2021.04.027

[99] Samardzic K , Rodgers KJ . Oxidised protein metabolism: recent insights. Biological Chemistry. 2017;398:1165‐1175. 28600903 10.1515/hsz-2017-0124

[100] Hazan (Ben‐Menachem) R , Lintzer D , Ziv T , et al. Mitochondrial‐derived vesicles retain membrane potential and contain a functional ATP synthase. EMBO Rep. 2023;24:e56114. 36929726 10.15252/embr.202256114 PMC10157309

[101] Mishra E , Thakur MK . Vitamin B(12)‐folic acid supplementation improves memory by altering mitochondrial dynamics, dendritic arborization, and neurodegeneration in old and amnesic male mice. J Nutr Biochem. 2024;124:109536. 37981108 10.1016/j.jnutbio.2023.109536

[102] Zhu L , Zhang Q , Hua C , Ci X . Melatonin alleviates particulate matter‐induced liver fibrosis by inhibiting ROS‐mediated mitophagy and inflammation via Nrf2 activation. Ecotoxicol Environ Saf. 2023;268:115717. 37992643 10.1016/j.ecoenv.2023.115717

[103] Goldberg MS , Fleming SM , Palacino JJ , et al. Parkin‐deficient mice exhibit nigrostriatal deficits but not loss of dopaminergic neurons. J Biol Chem. 2003;278:43628‐43635. 12930822 10.1074/jbc.M308947200

[104] Kitada T , Tong Y , Gautier CA , Shen J . Absence of nigral degeneration in aged parkin/DJ‐1/PINK1 triple knockout mice. J Neurochem. 2009;111:696‐702. 19694908 10.1111/j.1471-4159.2009.06350.x PMC2952933

[105] Mondal P , Towers C . Beyond mitophagy: mitochondrial‐derived vesicles can get the job done!. Autophagy. 2022;18:449‐451. 34781816 10.1080/15548627.2021.1999562 PMC8942527

[106] Picca A , Faitg J , Auwerx J , Ferrucci L , D'Amico D . Mitophagy in human health, ageing and disease. Nat Metab. 2023;5:2047‐2061. 38036770 10.1038/s42255-023-00930-8 PMC12159423

[107] Mishra S , Deep G . Mitochondria‐derived vesicles: potential nano‐batteries to recharge the cellular powerhouse. Extracell Vesicles Circ Nucl Acids. 2024;5:271‐275. 39092319 10.20517/evcna.2023.71 PMC11293460

[108] Li H , Gong W , Sun W , Yao Y , Han Y . Role of VPS39, a key tethering protein for endolysosomal trafficking and mitochondria‐lysosome crosstalk, in health and disease. J Cell Biochem. 2024;125(11):e30396. 36924104 10.1002/jcb.30396

[109] Harper JW , Ordureau A , Heo JM . Building and decoding ubiquitin chains for mitophagy. Nat Rev Mol Cell Biol. 2018;19:93‐108. 29358684 10.1038/nrm.2017.129

[110] Pickles S , Vigié P , Youle RJ . Mitophagy and quality control mechanisms in mitochondrial maintenance. Curr Biol. 2018;28:R170‐r185. 29462587 10.1016/j.cub.2018.01.004 PMC7255410

[111] Sheftel AD , Zhang AS , Brown C , Shirihai OS , Ponka P . Direct interorganellar transfer of iron from endosome to mitochondrion. Blood. 2007;110:125‐132. 17376890 10.1182/blood-2007-01-068148

[112] Dimitrov L , Lam SK , Schekman R . The role of the endoplasmic reticulum in peroxisome biogenesis. Cold Spring Harb Perspect Biol. 2013;5:a013243. 23637287 10.1101/cshperspect.a013243 PMC3632059

[113] Sugiura A , Mattie S , Prudent J , McBride HM . Newly born peroxisomes are a hybrid of mitochondrial and ER‐derived pre‐peroxisomes. Nature. 2017;542:251‐254. 28146471 10.1038/nature21375

[114] Phinney DG , Di Giuseppe M , Njah J , et al. Mesenchymal stem cells use extracellular vesicles to outsource mitophagy and shuttle microRNAs. Nat Commun. 2015;6:8472. 26442449 10.1038/ncomms9472 PMC4598952

[115] Liu D , Dong Z , Wang J , Tao Y , Sun X , Yao X . The existence and function of mitochondrial component in extracellular vesicles. Mitochondrion. 2020;54:122‐127. 32861876 10.1016/j.mito.2020.08.005

[116] Ferrucci L , Guerra F , Bucci C , Marzetti E , Picca A . Mitochondria break free: mitochondria‐derived vesicles in aging and associated conditions. Age Res Rev. 2024;102:102549. 10.1016/j.arr.2024.102549 PMC12155116 39427885

[117] Nicolás‐Ávila JA , Lechuga‐Vieco AV , Esteban‐Martínez L , et al. A network of macrophages supports mitochondrial homeostasis in the heart. Cell. 2020;183:94‐109 e123. 32937105 10.1016/j.cell.2020.08.031

[118] Yao W , Zhou J , Tang C , et al. Hydrogel microneedle patches loaded with stem cell mitochondria‐enriched microvesicles boost the chronic wound healing. ACS nano. 2024;18:26733‐26750. 39238258 10.1021/acsnano.4c06921 PMC11447894

[119] Hammood M , Craig AW , Leyton JV . Impact of endocytosis mechanisms for the receptors targeted by the currently approved antibody‐drug conjugates (ADCs) – a necessity for future ADC research and development. Pharmaceuticals (Basel). 2021;14. 10.3390/ph14070674 PMC8308841 34358100

[120] Miragoli M , Sanchez‐Alonso J , Bhargava A , et al. Microtubule‐dependent mitochondria alignment regulates calcium release in response to nanomechanical stimulus in heart myocytes. Cell Rep. 2016;14:140‐151. 26725114 10.1016/j.celrep.2015.12.014 PMC4983655

[121] Tu Y , Pal K , Austin J , Wang X . Filopodial adhesive force in discrete nodes revealed by integrin molecular tension imaging. Curr Biol. 2022;32:4386‐4396 e4383. 36084647 10.1016/j.cub.2022.08.040 PMC9613586

[122] Komori T , Kuwahara T . An update on the interplay between LRRK2, Rab GTPases and Parkinson's disease. Biomolecules. 2023;13. 10.3390/biom13111645 PMC10669493 38002327

[123] Zhao YG , Codogno P , Zhang H . Machinery, regulation and pathophysiological implications of autophagosome maturation. Nat Rev Mol Cell Biol. 2021;22:733‐750. 34302147 10.1038/s41580-021-00392-4 PMC8300085

[124] Torralba D , Baixauli F , Villarroya‐Beltri C , et al. Priming of dendritic cells by DNA‐containing extracellular vesicles from activated T cells through antigen‐driven contacts. Nat Commun. 2018;9:2658. 29985392 10.1038/s41467-018-05077-9 PMC6037695

[125] Cheng AN , Cheng L , Kuo C , et al. Mitochondrial Lon‐induced mtDNA leakage contributes to PD‐L1‐mediated immunoescape via STING‐IFN signaling and extracellular vesicles. J Immunother Cancer. 2020;8. 10.1136/jitc-2020-001372 PMC7713199 33268351

[126] Decout A , Katz JD , Venkatraman S , Ablasser A . The cGAS‐STING pathway as a therapeutic target in inflammatory diseases. Nat Rev Immunol. 2021;21:548‐569. 33833439 10.1038/s41577-021-00524-z PMC8029610

[127] Marchi S , Guilbaud E , Tait SWG , Yamazaki T , Galluzzi L . Mitochondrial control of inflammation. Nat Rev Immunol. 2023;23:159‐173. 35879417 10.1038/s41577-022-00760-x PMC9310369

[128] Chen L , Dong J , Liao S , et al. Loss of Sam50 in hepatocytes induces cardiolipin‐dependent mitochondrial membrane remodeling to trigger mtDNA release and liver injury. Hepatology. 2022;76:1389‐1408. 35313046 10.1002/hep.32471

[129] Ivashkiv LB , Donlin LT . Regulation of type I interferon responses. Nat Rev Immunol. 2014;14:36‐49. 24362405 10.1038/nri3581 PMC4084561

[130] Sahoo BR . Structure of fish Toll‐like receptors (TLR) and NOD‐like receptors (NLR). Int J Biol Macromol. 2020;161:1602‐1617. 32755705 10.1016/j.ijbiomac.2020.07.293 PMC7396143

[131] Fu R , Zhao L , Guo Y , et al. AIM2 inflammasome: a potential therapeutic target in ischemic stroke. Clin Immunol. 2024;259:109881. 38142900 10.1016/j.clim.2023.109881

[132] Lee S , Karki R , Wang Y , Nguyen LN , Kalathur RC , Kanneganti T . AIM2 forms a complex with pyrin and ZBP1 to drive PANoptosis and host defence. Nature. 2021;597:415‐419. 34471287 10.1038/s41586-021-03875-8 PMC8603942

[133] Lu A , Wu S , Niu J , et al. Aim2 couples With Ube2i for sumoylation‐mediated repression of interferon signatures in systemic lupus erythematosus. Arthritis Rheumatol. 2021;73:1467‐1477. 33559374 10.1002/art.41677 PMC8324518

[134] Zhang P , Liu Y , Hu L , et al. NLRC4 inflammasome‐dependent cell death occurs by a complementary series of three death pathways and determines lethality in mice. Sci Adv. 2021;7:eabi9471. 34678072 10.1126/sciadv.abi9471 PMC8535822

[135] Kong F , You H , Zheng K , Tang R , Zheng C . The crosstalk between pattern‐recognition receptor signaling and calcium signaling. Int J Biol Macromol. 2021;192:745‐756. 34634335 10.1016/j.ijbiomac.2021.10.014

[136] Li J , Xiao J , Gao M , et al. IRF/Type I IFN signaling serves as a valuable therapeutic target in the pathogenesis of inflammatory bowel disease. Int Immunopharmacol. 2021;92:107350. 33444921 10.1016/j.intimp.2020.107350

[137] Zhang L , Wei X , Wang Z , et al. NF‐kappaB activation enhances STING signaling by altering microtubule‐mediated STING trafficking. Cell Rep. 2023;42:112185. 36857187 10.1016/j.celrep.2023.112185

[138] Monzel AS , Enríquez JA , Picard M . Multifaceted mitochondria: moving mitochondrial science beyond function and dysfunction. Nat Metab. 2023;5:546‐562. 37100996 10.1038/s42255-023-00783-1 PMC10427836

[139] Bajwa E , Pointer CB , Klegeris A . The role of mitochondrial damage‐associated molecular patterns in chronic neuroinflammation. Mediators Inflamm. 2019;2019:4050796. 31065234 10.1155/2019/4050796 PMC6466851

[140] Schindler SM , Frank MG , Annis JL , Maier SF , Klegeris A . Pattern recognition receptors mediate pro‐inflammatory effects of extracellular mitochondrial transcription factor A (TFAM). Mol Cell Neurosci. 2018;89:71‐79. 29678518 10.1016/j.mcn.2018.04.005

[141] Wenzel TJ , Bajwa E , Klegeris A . Cytochrome c can be released into extracellular space and modulate functions of human astrocytes in a toll‐like receptor 4‐dependent manner. Biochim Biophys Acta Gen Subj. 2019;1863:129400. 31344401 10.1016/j.bbagen.2019.07.009

[142] Poillet‐Perez L , White E . MDVs to the rescue: how autophagy‐deficient cancer cells adapt to defective mitophagy. Dev Cell. 2021;56:2010‐2012. 34314695 10.1016/j.devcel.2021.06.022

[143] Tang M , Tu Y , Gong Y , et al. beta‐hydroxybutyrate facilitates mitochondrial‐derived vesicle biogenesis and improves mitochondrial functions. Molecular Cell. 2025;85:1395‐1410 e1395. 40118051 10.1016/j.molcel.2025.02.022

[144] Bao D , Zhao J , Zhou X , et al. Mitochondrial fission‐induced mtDNA stress promotes tumor‐associated macrophage infiltration and HCC progression. Oncogene. 2019;38:5007‐5020. 30894684 10.1038/s41388-019-0772-z PMC6755992

[145] Takenaga K , Koshikawa N , Nagase H . Intercellular transfer of mitochondrial DNA carrying metastasis‐enhancing pathogenic mutations from high‐ to low‐metastatic tumor cells and stromal cells via extracellular vesicles. BMC Mol Cell Biol. 2021;22:52. 34615464 10.1186/s12860-021-00391-5 PMC8496074

[146] Zhao F , Sun L , Yang N , et al. Increased release of microvesicles containing mitochondria is associated with the myeloid differentiation of AML‐M5 leukaemia cells. Exp Cell Res. 2020;395:112213. 32758487 10.1016/j.yexcr.2020.112213

[147] Abad E , Lyakhovich A . Movement of Mitochondria with mutant DNA through extracellular vesicles helps cancer cells acquire chemoresistance. ChemMedChem. 2022;17:e202100642. 34847299 10.1002/cmdc.202100642

[148] Salaud C , Alvarez‐Arenas A , Geraldo F , et al. Mitochondria transfer from tumor‐activated stromal cells (TASC) to primary glioblastoma cells. Biochem Biophys Res Commun. 2020;533:139‐147. 32943183 10.1016/j.bbrc.2020.08.101

[149] Hall AJ , Robertson AG , Hill LR , Rendina LM . Synthesis and tumour cell uptake studies of gadolinium(III)‐phosphonium complexes. Sci Rep. 2021;11:598. 33436690 10.1038/s41598-020-79893-9 PMC7804430

[150] Jiang H , Guo Y , Wei C , Hu P , Shi J . Nanocatalytic innate immunity activation by mitochondrial DNA oxidative damage for tumor‐specific therapy. Adv Mater. 2021;33:e2008065. 33797131 10.1002/adma.202008065

[151] Yang G , Pan Z , Zhang D , Cao Q , Ji L , Mao Z . Precisely assembled nanoparticles against cisplatin resistance via cancer‐specific targeting of mitochondria and imaging‐guided chemo‐photothermal therapy. ACS Appl Mater Interfaces. 2020;12:43444‐43455. 32883070 10.1021/acsami.0c12814

[152] Dickhout A , Koenen RR . Extracellular vesicles as biomarkers in cardiovascular disease; chances and risks. Front Cardiovasc Med. 2018;5:113. 30186839 10.3389/fcvm.2018.00113 PMC6113364

[153] Vasam G , Nadeau R , Cadete VJJ , Lavallée‐Adam M , Menzies KJ , Burelle Y . Proteomics characterization of mitochondrial‐derived vesicles under oxidative stress. FASEB J. 2021;35:e21278. 33769614 10.1096/fj.202002151R PMC8252493

[154] Zhao Q , Liu Z , Song P , Yuan Z , Zou MH . Mitochondria‐derived vesicle packaging as a novel therapeutic mechanism in pulmonary hypertension. Am J Respir Cell Mol Biol. 2024;70:39‐49. 37713305 10.1165/rcmb.2023-0010OC PMC10768832

[155] Chaiyarit S , Thongboonkerd V . Mitochondria‐derived vesicles and their potential roles in kidney stone disease. J Transl Med. 2023;21:294. 37131163 10.1186/s12967-023-04133-3 PMC10152607

